# Symmetry-Adapted Relaxation Theory (SART): Variational Embedding for Convergent Infinite-Order Induction without Overpolarization

**DOI:** 10.1021/acs.jctc.6c00770

**Published:** 2026-08-13

**Authors:** Humahuti Dihingia, Bartosz Tyrcha, Edoardo Vanich, Konrad Patkowski, Alston J. Misquitta, Piotr S. Żuchowski

**Affiliations:** † Institute of Physics, 317747 Faculty of Physics, Astronomy and Informatics, Nicolaus Copernicus University in Toruń, Grudzia̧dzka 5/7, 87-100 Toruń, Poland; ‡ 27063 Sorbonne Université, Laboratoire de Chimie Théorique, UMR 7616 CNRS, 75005 Paris, France; ¶ Department of Chemistry and Biochemistry, 1383 Auburn University, Auburn, Alabama 36849, United States; § Department of Physics and Astronomy, Queen Mary University of London, London E1 4NS, United Kingdom

## Abstract

The induction part of intermolecular interaction energy describes the effect of the mutual polarization of subsystems. Low-order induction effects can be reasonably described by symmetry-adapted perturbation theory (SAPT), but the capture of important higher-order polarization effects requires the use of an external correction from supermolecular Hartree–Fock (HF) theory, which is not free from artifacts. When one describes induction through a response to an embedding potential representing the other molecule(s) (which is the case in a number of existing approaches such as the electrostatic embedding, Hartree–Hartree–Fock, and explicit polarization methods), it is easy to succumb to overpolarization unless the embedding potential fully accounts for the exchange effects, enforcing the Pauli exclusion principle and preventing a variational collapse to a Pauli-forbidden state. Here, we propose a new embedding framework that accounts for both electrostatic polarization and exchange effects in many-body systems. As a proof of principle, we apply the novel embedding potentials in a variational approach called symmetry-adapted relaxation theory (SART) that succeeds in recovering infinite-order induction energy from the HF method without a need to compute the HF wave function or energy of the entire complex. SART is expected to be the foundation for a new class of intermolecular perturbation theories, while the newly proposed potentials can also be applied to incorporate complete exchange into various embedding algorithms.

## Graphical abstract

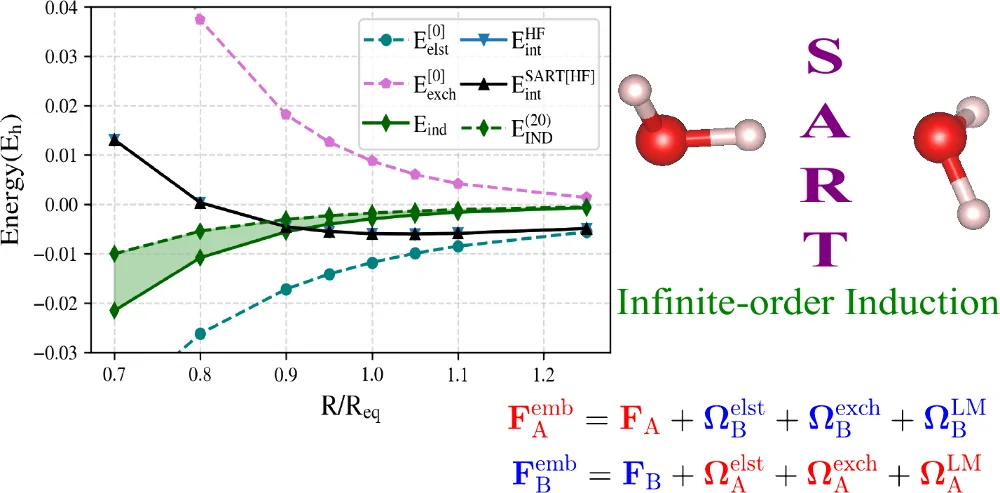

## 1 Introduction

Intermolecular interactions are orders of magnitude weaker than typical covalent forces, yet they play a crucial role in determining the structure of molecular crystals, the thermodynamic behavior of gases, the spectra of molecular complexes, and even the course of chemical reactions. As the demand for accurate predictions of interaction potentials continues to grow, there is a pressing need for new methods that offer an improved and physically grounded description of interaction energies.

Symmetry-Adapted Perturbation Theory (SAPT)^1–3^ has become firmly established as one of the most reliable methods for obtaining interaction energies at a favorable cost-to-accuracy ratio, at the same time being a powerful tool for energy decomposition into physically meaningful quantities. Conceptually, SAPT relies on the partitioning of the Hamiltonian into monomers A and B, with Hamiltonians *H*_A_ and *H*_B_, with the interactions between them described by the perturbation *V*,

1 $$H=H_{A}+H_{B}+\lambda V$$

and performing the Rayleigh–Schrödinger (RS) perturbation expansion in powers of λ. To satisfy the Pauli exclusion principle between electrons of the different subsystems, it is essential to perform an appropriate symmetry forcing scheme, which is usually applied only to the energy expressions within the symmetrized Rayleigh–Schrödinger (SRS) approach.^1^

Nearly two decades ago, the strong position of SAPT was reinforced by the demonstration that using asymptotically corrected^4^ density functional theory (DFT) for the monomers can significantly improve the description of first-order SAPT corrections, while the second-order induction and dispersion interactions^5^ can be obtained with frequency-dependent density susceptibility functions computed using time-dependent DFT theory.^6–8^ This variant, known as SAPT­(DFT), has since become a standard tool for the computation of interaction energies. The applicability of SAPT and SAPT­(DFT) now spans systems ranging from small and medium-sized molecules^9^ to large complexes containing 100–200 atoms.^10–13^ Moreover, SAPT has been extended to treat both open-shell single-reference 14,15 and multiconfigurational systems.^16–18^ In numerous benchmark studies, the most commonly used SAPT­(DFT) variant has demonstrated accuracy comparable to that of gold-standard quantum chemistry methods,^19–21^ while maintaining a more favorable computational scaling: *N* ^5^ with respect to basis set size *N*, compared to *N* ^7^ for the “gold standard” supermolecular coupled-cluster approach with single, double, and perturbative triple excitations, CCSD­(T).

However, one major unresolved problem of SAPT and SAPT­(DFT) is the accurate description of induction and exchange effects beyond the second order. The SAPT series cannot be simply truncated at the second order: in systems with ions, polar molecules or highly polarizable species, such a truncation can lead to significant errors, and even the repulsive wall of the potential may not be reproduced qualitatively.^22^ Third-order SAPT corrections were derived and implemented by Patkowski and co-workers,^23–25^ but these corrections often perform poorly for polar systems, and including the third-order induction and exchange-induction terms can sometimes worsen the results. A commonly used approximation to the SAPT exchange corrections (termed *S* ^2^ with *S* denoting the overlap matrix between monomers), which limits the symmetry forcing operator to exchanges of pairs of electrons, often breaks down for the description of repulsive walls, especially at third order, as the induction and exchange induction should be treated on equal footing.^24,26–28^ A common remedy to account for higher-order interaction terms is to apply a supermolecular approach as an approximate treatment of third- and higher-order induction effects. Since the first-order electrostatic and exchange, and second-order induction and exchange induction are included in the supermolecular Hartree–Fock interaction energy *E*_int_^HF^, the remainder δ_int_^HF^ can be written using the standard SAPT notation for corrections *E* ^(*mn*)^ of order *m* in intermolecular interaction and *n* in the intramonomer correlation:^29^

2 $$\delta_{\mathrm{int}}^{\mathrm{HF}}=E_{\mathrm{int}}^{\mathrm{HF}}-\left[E_{\mathrm{elst}}^{\left(10\right)}+E_{\mathrm{exch}}^{\left(10\right)}+E_{\mathrm{ind},\mathrm{resp}}^{\left(20\right)}+E_{\mathrm{exch}-\mathrm{ind},\mathrm{resp}}^{\left(20\right)}\right]$$

where *E*_elst_^(10)^, *E*_exch_^(10)^ are the first-order electrostatic and exchange energies, *E*_ind,resp_^(20)^ and *E*_exch–ind,resp_^(20)^ are the second-order induction and exchange-induction contributions, all from SAPT0, and the “resp” label in case of induction denotes orbital relaxation in the field of interacting partner. The δ_int_^HF^ term also includes some subtle effects called the Murrell and Landshoff terms, Δ_LM_ which are zeroth order in the interaction operator. For the determinantal wave function, Δ_LM_ can be expressed as^30^ the difference between the Heitler–London energy and the first-order SAPT value.

Because δ_int_^HF^ is a supermolecular correction, it inherits all of the disadvantages of that approach: a lack of physical interpretability, the need to correct for the basis-set superposition error (BSSE), no connection to monomer properties, and no rigorous decomposition into fragment–fragment contributions for a fine-grained analysis.^3,10^ It also does not include intramonomer electron correlation in any fixable way. Importantly, in some exotic cases, no obvious analogue of δ_int_^HF^ exists—for example, in multiconfigurational SAPT or in certain systems for which Hartree–Fock calculations are impossible, such as the interaction potentials of systems undergoing Penning ionization.^31^ From a pragmatic point of view, δ_int_^HF^ leads to systematic overbinding. A notable example system where such overbinding takes place is the benzene dimer.^8,32^ The δ_int_^HF^ correction was used in all of the SAPT levels benchmarked in refs 19 and 33. Indeed, statistical overbinding was observed in most of the studied SAPT cases. Finally, in the extensive benchmark sets investigated by Masumian and Boese,^20^ severe problems with δ_int_^HF^ were detected for the systems with heavy atoms.

To extend SAPT into new and difficult areas, the major challenge is accounting for induction energy beyond second order. Thus, new solutions are needed which, given problems with the SAPT convergence, are based on a variational approach, such as the one introduced by Gniewek and Jeziorski for the exchange splitting.^34^ A new formulation of the infinite-order induction problem should be designed in such a way that it is possible to adapt it to more than two interacting systems. Indeed, induction is inherently a many-body effect, one of the main driving forces behind the nonadditive effects in clusters.^35,36^ Several approaches have been proposed that partially address some of these requirements. In Sadlej’s Hartree–Hartree–Fock approach,^37^ induction—without exchange—was incorporated variationally by solving the Hartree–Fock equations in the field of the partner fragments. However, such an approach works only for a large separation between monomers and eventually leads to the overpolarization catastrophe: the escape of electrons toward the attracting potential of the partner.

To prevent such an overpolarization, Adams’s modified perturbation scheme ZI-SAPT^38^ introduced a new Hamiltonian partitioning that incorporated the electric field of the interacting partner into the zeroth-order Hamiltonian and treated the remainder as a perturbation. Importantly, in the ZI approach, the attractive singularities in the electrostatic potential were weakened using an empirical regularization to prevent tunneling of electrons between interacting partners and discourage overpolarization. Building on the ZI framework, Patkowski et al. examined the Li ··· H complex, which is weakly bound in the triplet state but forms a covalent bond in the singlet. Using regularization of the electrostatic potential together with strong (beyond-SRS) symmetry forcing, they reproduced both the covalent singlet bond and the shallow triplet state.^39,40^

More recently, Herbert and co-workers introduced XSAPT,^41^ which builds on the X-Pol method of Gao and co-workers.^42^ In X-Pol, the monomer Hamiltonians are augmented by point charges representing the interacting partners, and the resulting Hartree–Fock or Kohn–Sham equations are solved one subsystem at a time until self-consistency is achieved between the point charges in the Hamiltonian and the point charges resulting from the converged HF or KS density.^41^ Because the embedding potential consists of point charges only, with no exchange component, XSAPT is in principle susceptible to overpolarization. In practice, this is mitigated by the use of monomer-centered basis sets (the absolutely localized molecular orbital (ALMO) approach), which effectively confines the monomer orbitals and prevents unphysical charge transfer. However, this protection vanishes as the basis set approaches completeness, and XSAPT has no well-defined complete basis set limit for the induction energy.

On a fundamental level, the computation of infinite-order induction shares the same goal with quantum embedding theories: determining a subsystem’s electronic structure self-consistently in the potential of its environment. In frozen-density embedding,^43,44^ subsystem interactions are mediated through a nonadditive kinetic energy functional, which remains approximate. Projection-based embedding schemes^45–47^ resolve this by enforcing intersubsystem orthogonality through a level-shift operator, making the embedding in principle exact. However, the orthogonalization destroys the identity of individual monomers, precluding a clean connection to SAPT-like energy decomposition or monomer-based perturbation theory.

The overpolarization problem in embedding theories has a novel resolution in a class of nonorthogonal theories starting with SCF-MI,^48^ which led to BLW,^49^ and later to X-Pol-X^50^ and fragment exchange potential^51^ and related methods. These formulations, which are the closest in relation to what we will do here, include explicit consideration of intermonomer exchange, however this is done with a block-diagonal approximation on the molecular orbitals, which excludes the possibility of charge transfer. Consequently, while the exchange is well-defined by these methods, the induction energy is underestimated due to the missing charge transfer contribution which is known to be strong in systems, for example, with hydrogen bonds.

Gutowski and Piela^52^ introduced a Pauli Blockade approach, later adapted to the density functional formalism by Rajchel and co-workers.^53–55^ While this approach incorporates exchange through orthogonalized orbitals, the interaction energy is still obtained by subtracting monomer energies from a dimer energy expressed in mixed, orthogonalized orbitals—making it effectively supermolecular, with all the associated disadvantages.

Motivated by this challenge, we introduce here a novel embedding scheme rooted in SAPT, in which monomer Hamiltonians are augmented by potentials representing the electrostatic field, Pauli exchange repulsion, and additional contributions arising from the nonorthogonality of subsystem orbitals. The latter prove essential for maintaining the variational bound on the total energy of the system and for preserving the Hamiltonian symmetry. The method employs a trial wave function that is the antisymmetrized product of the monomer wave functions; although very general, the theory is first presented here for monomers described by Hartree–Fock determinants. The variational principle leads to self-consistent field equations in which monomer orbitals relax in the field of the interacting partners—hence the name Symmetry-Adapted Relaxation Theory (SART). The resulting method reproduces the supermolecular Hartree–Fock interaction energy exactly, but without *ever* computing the supermolecular wave function. While the exact recovery of the Hartree–Fock interaction energy validates the approach, of greater consequence are the relaxed monomer orbitals “dressed” by induction and quenched by exchange—as these form a natural starting point for a SAPT-like perturbation theory based on relaxed monomers, in particular, for the calculation of dispersion interaction and the induction-dispersion coupling.

We have structured this paper in the following manner. First, in Section 2 we introduce the formalism and focus on the variational principle and Ansatz. Then, we present the derivation of the novel embedding potentials which are used to perturb the Hartree–Fock equations for the subsystem. Section 3 describes the details of the numerical implementation of the SART method. We will discuss the nature of the newly derived terms and present preliminary results for a few model systems in Section 4, proving that our method exactly reproduces the induction effects for the HF case. Finally, Section 5 concludes our paper with a summary and an outlook.

## 2 Theory

We would first like to address the notation and some important tools used in the present paper. For molecular orbital and spin–orbital indices, we follow the conventions established in refs 2 and 56–60 . Greek letters designate spin–orbital indices, while Latin letters represent molecular orbital indices. Specifically, the unoccupied spin–orbitals of the monomer A are designated as ρ, ρ_1_, ρ_2_, ..., and those of monomer B are labeled as σ, σ_1_, σ_2_, ... The occupied spin–orbitals are indicated by α, α_1_, α_2_, ... for monomer A and β, β_1_, β_2_, ... for monomer B. We use λ, λ_1_, λ_2_, ... and κ, κ_1_, κ_2_, ... to represent the general spin–orbital indices of monomer A, and μ, μ_1_, μ_2_, ... and ν, ν_1_, ν_2_, ... for monomer B. We work primarily in the spin–orbital representations, but we provide the final formulas in terms of molecular orbitals for the spin-restricted case. The Einstein summation convention for repeated indices is used throughout the present paper.

We employ the framework established in refs 29 and 57, which is constructed within the product Fock space $F_{A}⊗F_{B}$ . This space forms the basis for a second quantization formalism of SAPT exchange energies. Within the context of SAPT, which uses two separate Fock spaces for individual monomers, we introduce two unique sets of creation and annihilation operators, *a*_λ_^†^, *a*_λ_ in $F_{A}$ and *b*_μ_^†^, *b*_μ_ in $F_{B}$ . These operators obey the standard anticommutation relations within their respective spaces and commute between the two sets. We also introduce the *n*-particle spin–orbital substitution operators

3 $$a_{\kappa_{1}\kappa_{2}...\kappa_{n}}^{\lambda_{1}\lambda_{2}...\lambda_{n}}=a_{\lambda_{1}}^{†}a_{\lambda_{2}}^{†}...a_{\lambda_{n}}^{†}a_{\kappa_{n}}...a_{\kappa_{2}}a_{\kappa_{1}}$$

4 $$b_{\nu_{1}\nu_{2}...\nu_{n}}^{\mu_{1}\mu_{2}...\mu_{n}}=b_{\mu_{1}}^{†}b_{\mu_{2}}^{†}...b_{\mu_{n}}^{†}b_{\nu_{n}}...b_{\nu_{2}}b_{\nu_{1}}$$

In particular, the operators *a*_α_^ρ^ and *b*_β_^σ^ represent single excitations out of the reference Slater determinant. Throughout this paper, Φ_A_ and Φ_B_ denote Slater determinants built from orbitals varied in the field of the partner molecules.

### 2.1 Variational Functional for the Dimer

To proceed, let us employ the variational principle for the dimer, written in its most general form as

5 $$E_{0}=\underset{\Psi_{\mathrm{AB}}}{\min}E^{\mathrm{AB}}\left[\Psi_{\mathrm{AB}}\right],E^{\mathrm{AB}}\left[\Psi_{\mathrm{AB}}\right]=\frac{\left\langle \Psi_{\mathrm{AB}}\right|H_{A}+H_{B}+V\left|\Psi_{\mathrm{AB}}\right\rangle }{\left\langle \Psi_{\mathrm{AB}}\right|\Psi_{\mathrm{AB}}\rangle }$$

We seek a stationary point of *E* ^AB^[Ψ_AB_] with respect to variations of the trial function Ψ_AB_. What class of variations suffices to recover *exclusively* the induction contribution to the interaction energy? The induction arises from single excitations (and de-excitations) on one monomer at a time.^25,29,61^ A general expression for the wave function of the dimer which accounts for induction energy can therefore be taken as Ψ_AB_ = *U*_A_ Ψ_A_ *U*_B_ Ψ_B_, where the unitary operators *U*_A_ and *U*_B_ act independently on the monomer wave functions Ψ_A_ and Ψ_B_. Such an Ansatz does not account for the dispersion interaction, for which there is a requirement of excitations/de-excitations on both monomers simultaneously. The simplest possible trial wave function, which we will use here, is composed of single Slater determinants on each monomer Φ_A_, Φ_B_. The trial wave function must also be antisymmetric with respect to exchanges of electrons between monomers. Thus, in the spirit of SAPT, we use the antisymmetrizer A to ensure such symmetry adaptation.

We now discuss how to enforce antisymmetry, which is key to properly account for the Pauli exclusion principle. Using the commutation relation $\left[H_{A}+H_{B}+V,A\right]=0$ , and the idempotency $A^{2}=A$ , the functional of eq 5 can be written as

6 $$E^{\mathrm{AB}}\left[\Phi_{A},\Phi_{B}\right]=\frac{\left\langle \Phi_{A}\Phi_{B}\right|\left(H_{A}+H_{B}+V\right)A\left|\Phi_{A}\Phi_{B}\right\rangle }{\left\langle \Phi_{A}\Phi_{B}\right|A\left|\Phi_{A}\Phi_{B}\right\rangle }$$

In order to separate the polarization and exchange effects, let us decompose the antisymmetrizer as

7 $$A=\frac{N_{A}!N_{B}!}{\left(N_{A}+N_{B}\right)!}A_{A}A_{B}\left(1+P\right)$$

where $A_{A}$ and $A_{B}$ are antisymmetrizers acting within the spaces of monomers A and B, respectively, and *N*_A_ and *N*_B_ represent the number of electrons associated with each monomer.^57^ The operator of the intersystem permutations between A and B, P , has a proper second-quantized representation,^60^ which requires the dimer-centered basis set. In such a basis, excitations from monomer A to orbitals localized on B, and vice versa, are possible. Thus, this trial wave function naturally captures charge-transfer effects.

In our recent works on exchange energy, we conjectured^60,62^ an analogue of the linked–cluster theorem that eliminates the denominator by retaining only the linked part of the numerator. More recently, Cieslinski and collaborators have rigorously proven our conjecture for the first-order SAPT energy utilizing arguments from graph theory.^63^ Hence, we can rewrite eq 6 as a sum of linked terms:

8 $$E^{\mathrm{AB}}\left[\Phi_{A},\Phi_{B}\right]=\left\langle \Phi_{A}\Phi_{B}\right|H_{A}\left|\Phi_{A}\Phi_{B}\right\rangle +\left\langle \Phi_{A}\Phi_{B}\right|H_{B}\left|\Phi_{A}\Phi_{B}\right\rangle +\left\langle \Phi_{A}\Phi_{B}\right|V\left|\Phi_{A}\Phi_{B}\right\rangle +\left\langle \Phi_{A}\Phi_{B}\right|\left(H_{A}+H_{B}\right)P|\Phi_{A}\Phi_{B}\rangle_{L}+\left\langle \Phi_{A}\Phi_{B}\right|VP|\Phi_{A}\Phi_{B}\rangle_{L}$$

The first three terms in eq 8 contain only linked contributions because they are each expectation values of a single operator. The products of operators in the last two terms, i.e., $\left(H_{A}+H_{B}\right)P$ and VP , are not Hermitian and this would later lead to non-Hermitian contributions to the SART Fock matrix, which is undesirable. It is convenient to recast the P -containing contributions so that each term is Hermitian (and thus represented by a symmetric matrix in the finite-basis implementation). To this end, let us notice that

(HA+HB+V)A=A(HA+HB+V)=12((HA+HB+V)A+A(HA+HB+V)) 9

Using the above equality in the numerator of eq 6, the functional can be rewritten as

10 $$E^{\mathrm{AB}}\left[\Phi_{A},\Phi_{B}\right]=\left\langle \Phi_{A}\Phi_{B}\right|H_{A}\left|\Phi_{A}\Phi_{B}\right\rangle +\left\langle \Phi_{A}\Phi_{B}\right|H_{B}\left|\Phi_{A}\Phi_{B}\right\rangle +\left\langle \Phi_{A}\Phi_{B}\right|V\left|\Phi_{A}\Phi_{B}\right\rangle +\frac{1}{2}\left\langle \Phi_{A}\Phi_{B}\right|\left(H_{A}+H_{B}\right)P+P\left(H_{A}+H_{B}\right)\left|\Phi_{A}\Phi_{B}\rangle_{L}+\frac{1}{2}\left\langle \Phi_{A}\Phi_{B}\right|VP+PV\right|\Phi_{A}\Phi_{B}\rangle_{L}$$

For convenience, from now on, let us use the anticommutator symbol for the symmetrized sum {X,P}=XP+PX .

We can evaluate the interaction energy as the difference between the dimer and monomer energies,

11 $$E_{\mathrm{int}}^{\mathrm{AB}}=E^{\mathrm{AB}}\left[\Phi_{A},\Phi_{B}\right]-E_{A}-E_{B}$$

However, one should note that *E*_int_^AB^ can be expressed solely via operators acting in the product Hilbert space of $H_{A}⊗H_{B}$ , unlike in the supermolecular method, which acts in the $H_{\mathrm{AB}}$ space—the Hilbert space of the dimer.

We now briefly interpret each contribution in eq 10. The terms ⟨Φ_A_Φ_B_|*H*_A_|Φ_A_Φ_B_⟩ and ⟨Φ_A_Φ_B_|*H*_B_|Φ_A_Φ_B_⟩ collect the monomer Hamiltonians evaluated in the *relaxed* orbitals, i.e., orbitals optimized for A and B in the presence of their partners, separately. The difference between these quantities and the isolated monomer energies *E*_A_ and *E*_B_ corresponds to the apparent deformation energy, which we formally introduce as

12 $$\begin{array}{c} \Delta E_{A}=\left\langle \Phi_{A}\Phi_{B}\right|H_{A}\left|\Phi_{A}\Phi_{B}\right\rangle -E_{A}\\ \Delta E_{B}=\left\langle \Phi_{A}\Phi_{B}\right|H_{B}\left|\Phi_{A}\Phi_{B}\right\rangle -E_{B} \end{array}$$

These are analogous to those discussed in related schemes, such as the Pauli Blockade method.^52–54^ A similar quantity, termed the electronic preparation energy, arises in the local energy decomposition of Bistoni and co-workers.^64,65^ ⟨Φ_A_Φ_B_|*V*|Φ_A_Φ_B_⟩ represents the electrostatic interaction evaluated in the relaxed orbitals (*E*_elst_); in the absence of orbital relaxation this reduces to the standard SAPT electrostatic energy. The following term,

13 $$E_{\mathrm{LM}}=\frac{1}{2}\left\langle \Phi_{A}\Phi_{B}\right|\left\{H_{A}+H_{B},P\right\}|\Phi_{A}\Phi_{B}\rangle_{L}$$

is of zeroth leading order in the interaction *V* and arises from antisymmetrization; in the case of the canonical orbitals of unperturbed (isolated) monomers, *E*_LM_ can be identified with the sum of the Landshoff and Murrell “delta” terms included in the Heitler–London interaction energy for determinant wave functions.^30,66,67^ However, these terms were never discussed for correlated wave functions. The decomposition into the Landshoff and Murrell terms comes from the Møller–Plesset partitioning *H*_A_ = *F*_A_+*W*_A_ and *H*_B_ = *F*_B_ + *W*_B_, which allows us to define

14 $$\begin{array}{l} E_{L}=\frac{1}{2}\left\langle \Phi_{A}\Phi_{B}\right|\left\{\left(F_{A}+F_{B}\right),P\right\}|\Phi_{A}\Phi_{B}\rangle_{L}\\ E_{M}=\frac{1}{2}\left\langle \Phi_{A}\Phi_{B}\right|\left\{\left(W_{A}+W_{B}\right),P\right\}|\Phi_{A}\Phi_{B}\rangle_{L} \end{array}$$

Note that, for unperturbed orbitals, *E*_LM_ is the difference between the SAPT first-order interaction energy and the variational Heitler–London energy. For such a case, *E*_L_ is zero and *E*_M_ is proportional to *S* ^4^. Finally, we denote *E*_exch_ = $\left\langle \Phi_{A}\Phi_{B}\right|VP|\Phi_{A}\Phi_{B}\rangle_{L}$ , which is the *relaxed* exchange energy. Hence, the total interaction energy can be expressed as the sum of these primary components,

15 $$E_{\mathrm{int}}^{\mathrm{AB}}=\Delta E_{A}+\Delta E_{B}+E_{\mathrm{LM}}+E_{\mathrm{elst}}+E_{\mathrm{exch}}$$

### 2.2 Generalized Brillouin Condition for the Dimer: Effective Potentials of Subsystems

The stationary point of the bifunctional *E* ^AB^ is obtained by parametrizing each monomer’s orbitals through anti-Hermitian rotation operators *C*_A_ and *C*_B_, which act exclusively within the orbital spaces of monomers A and B:

16 $$C_{A}^{†}=-C_{A},C_{B}^{†}=-C_{B}$$

Following the standard occupied–virtual parametrization, we write *C*_A_ = κ_A_ – κ_A_^†^ with κ_A_ = (κ_A_)_ρ_^α^ *a*_α_^ρ^ and, analogously, *C*_B_ = κ_B_ – κ_B_^†^ with κ_B_ = (κ_B_)_σ_^β^ *b*_β_^σ^. The orbitals are then rotated according to *U* = *e* ^*C*_A_^ *e* ^*C*_B_^ = *e* ^*C*_A_+*C*_B_^, and stationarity is imposed by requiring that the first variation of *E* ^AB^ with respect to *C*_A_ and *C*_B_ vanishes. For such exponential parametrizations, it is well-known (Thouless,^68^ Kutzelnigg,^69,70^ Lévy–Berthier,^71^ and, in the MCSCF context, refs 72–74 ) that the stationary condition is equivalent to the generalized Brillouin condition, which, stated for a generic Slater determinant Φ, reads ⟨Φ|[*H*, *C*]|Φ⟩ = 0 for all anti-Hermitian *C*. For a single Slater determinant and occupied–virtual excitations, this reduces to the vanishing of the occupied–virtual Fock matrix block (*F*_αρ_ = 0).

In the case of our functional, eq 10, the stationarity of *E* ^AB^ is achieved for Φ_A_, Φ_B_ when^69^

$$\langle \Phi_{A}\Phi_{B}|e^{-C_{A}-C_{B}}\left(H_{A}+H_{B}+V+\frac{1}{2}\left\{H_{A}+H_{B}+V,P\right\}\right)e^{C_{A}+C_{B}}|\Phi_{A}\Phi_{B}\rangle_{L}-{\left\langle \Phi_{A}\Phi_{B}|(H_{A}+H_{B}+V+\frac{1}{2}\left\{H_{A}+H_{B}+V,P\right\})|\Phi_{A}\Phi_{B}\right\rangle }_{L}=O\left(C_{A}^{2}\right)+O\left(C_{B}^{2}\right)+O\left(C_{A}C_{B}\right)$$ 17

for arbitrary *C*_A_, *C*_B_. Now, using the Baker–Campbell–Hausdorff expansion of the first matrix element and retaining only terms linear in *C*_A_ or *C*_B_, one obtains

18 $${\langle \Phi_{A}\Phi_{B}|[H_{A}+H_{B}+V+\frac{1}{2}\left\{H_{A}+H_{B}+V,P\right\},C_{A}+C_{B}]|\Phi_{A}\Phi_{B}\rangle }_{L}=0$$

which decomposes into the following two conditions:

19 $$0=\left\langle \Phi_{A}\Phi_{B}\right|\left[H_{A},C_{A}\right]\left|\Phi_{A}\Phi_{B}\right\rangle +\left\langle \Phi_{A}\Phi_{B}\right|\left[V,C_{A}\right]\left|\Phi_{A}\Phi_{B}\right\rangle +\left\langle \Phi_{A}\Phi_{B}\right|\left[\frac{1}{2}\left\{H_{A}+H_{B},P\right\},C_{A}\right]\left|\Phi_{A}\Phi_{B}\rangle_{L}+\left\langle \Phi_{A}\Phi_{B}\right|\left[\frac{1}{2}\left\{V,P\right\},C_{A}\right]\right|\Phi_{A}\Phi_{B}\rangle_{L}$$

20 $$0=\left\langle \Phi_{A}\Phi_{B}\right|\left[H_{B},C_{B}\right]\left|\Phi_{A}\Phi_{B}\right\rangle +\left\langle \Phi_{A}\Phi_{B}\right|\left[V,C_{B}\right]\left|\Phi_{A}\Phi_{B}\right\rangle +\left\langle \Phi_{A}\Phi_{B}\right|\left[\frac{1}{2}\left\{H_{A}+H_{B},P\right\},C_{B}\right]\left|\Phi_{A}\Phi_{B}\rangle_{L}+\left\langle \Phi_{A}\Phi_{B}\right|\left[\frac{1}{2}\left\{V,P\right\},C_{B}\right]\right|\Phi_{A}\Phi_{B}\rangle_{L}$$

To simplify the above equations, we have used the fact that operators depending on distinct coordinates commute (for example, *H*_A_ and *C*_B_), and each of the matrix elements present in the above equation is symmetric and can be decomposed into parts related to excitations and de-excitations, which are Hermitian conjugates of each other: ⟨Φ_A_Φ_B_|[*X*, *a*_α_^ρ^]|Φ_A_Φ_B_⟩ = – ⟨Φ_A_Φ_B_|[*X*, *a*_ρ_^α^]|Φ_A_Φ_B_⟩*, for *X* ^†^ = *X*.

Since these equations vanish for arbitrary variations, the matrix elements must satisfy the following conditions:

21 $$\left\langle \Phi_{A}\Phi_{B}\right|\left[H_{A},a_{\alpha }^{\rho }\right]\left|\Phi_{A}\Phi_{B}\right\rangle +\left\langle \Phi_{A}\Phi_{B}\right|\left[V,a_{\alpha }^{\rho }\right]\left|\Phi_{A}\Phi_{B}\right\rangle +\left\langle \Phi_{A}\Phi_{B}\right|\left[\frac{1}{2}\left\{H_{A}+H_{B},P\right\},a_{\alpha }^{\rho }\right]\left|\Phi_{A}\Phi_{B}\rangle_{L}+\left\langle \Phi_{A}\Phi_{B}\right|\left[\frac{1}{2}\left\{V,P\right\},a_{\alpha }^{\rho }\right]\right|\Phi_{A}\Phi_{B}\rangle_{L}=0$$

and

22 $$\left\langle \Phi_{A}\Phi_{B}\right|\left[H_{B},b_{\beta }^{\sigma }\right]\left|\Phi_{A}\Phi_{B}\right\rangle +\left\langle \Phi_{A}\Phi_{B}\right|\left[V,b_{\beta }^{\sigma }\right]\left|\Phi_{A}\Phi_{B}\right\rangle +\left\langle \Phi_{A}\Phi_{B}\right|\left[\frac{1}{2}\left\{H_{A}+H_{B},P\right\},b_{\beta }^{\sigma }\right]\left|\Phi_{A}\Phi_{B}\rangle_{L}+\left\langle \Phi_{A}\Phi_{B}\right|\left[\frac{1}{2}\left\{V,P\right\},b_{\beta }^{\sigma }\right]\right|\Phi_{A}\Phi_{B}\rangle_{L}=0$$

Using the fact that (*F*_A_)_α_^ρ^ ≡ ⟨Φ_A_Φ_B_|[*H*_A_, *a*_α_^ρ^]|Φ_A_Φ_B_⟩ and (*F*_B_)_β_^σ^ ≡ ⟨Φ_A_Φ_B_|[*H*_B_, *b*_β_^σ^]|Φ_A_Φ_*B*_⟩ are the Fock matrix elements of the isolated monomers A and B, respectively, let us inspect the remaining terms in eqs 21 and 22 to clarify their origin and physical meaning. The following matrix elements can be identified:

- ⟨Φ_A_Φ_B_|[*V*, *b*_β_^σ^]|Φ_A_Φ_B_⟩ = (*v*_A_)_β_^σ^ + *v*_βα_^σα^ ≡ (Ω_A_^elst^)_β_^σ^ are matrix elements of the electrostatic potential resulting from subsystem A (where *v*_A_ denotes the electron-nuclei interaction potential due to subsystem A), and, analogously, (Ω_B_^elst^)_α_^ρ^ are identified from ⟨Φ_A_Φ_B_|[*V*, *a*_α_^ρ^]|Φ_A_Φ_B_⟩.
- The terms $\left\langle \Phi_{A}\Phi_{B}\right|\left[\frac{1}{2}\left\{V,P\right\},b_{\beta }^{\sigma }\right]|\Phi_{A}\Phi_{B}\rangle_{L}$ and $\left\langle \Phi_{A}\Phi_{B}\right|\left[\frac{1}{2}\left\{V,P\right\},a_{\alpha }^{\rho }\right]|\Phi_{A}\Phi_{B}\rangle_{L}$ represent the *Pauli exchange* analogue of the electrostatic potential. Although not usually highlighted out explicitly in this form, closely related expressions appear in the derivation of the second-order exchange–induction energy without the *S* ^2^ approximation by Schäffer and Jansen.^75^ These define off-diagonal blocks of certain nonlocal potentials, which we denote by Ω_A_^exch^ and Ω_B_^exch^, respectively.
- The terms $\left\langle \Phi_{A}\Phi_{B}\right|\left[\frac{1}{2}\left\{H_{A}+H_{B},P\right\},b_{\beta }^{\sigma }\right]|\Phi_{A}\Phi_{B}\rangle_{L}$ and $\left\langle \Phi_{A}\Phi_{B}\right|\left[\frac{1}{2}\left\{H_{A}+H_{B},P\right\},a_{\alpha }^{\rho }\right]|\Phi_{A}\Phi_{B}\rangle_{L}$ are the orbital-rotation derivatives of the *E*_LM_ contributions. They are essential to preserve the variational character of the bifunctional *E* ^AB^ and enforce symmetry adaptation of the monomer wave functions. As we stated previously, these terms are of zeroth leading-order in the interaction operator *V*. We refer to these as the Landshoff–Murrell potentials, Ω_A_^LM^ and Ω_B_^LM^. While the *E*_LM_ energy terms are small, and for unperturbed monomers vanish exponentially with distance similar to *S* ^4^, which is faster than the exchange energy, the leading term of the LM potentials is proportional to *S* ^2^ and turns out to be essential for the convergence of the SCF equations.

Equations 21 and 22 define the *embedding* for the Fock matrices of monomers in the complete, exchange-aware field of the interacting partner:

23 $$\begin{array}{c} F_{A}^{\mathrm{emb}}=F_{A}+\Omega_{B}^{\mathrm{elst}}+\Omega_{B}^{\mathrm{exch}}+\Omega_{B}^{\mathrm{LM}}\\ F_{B}^{\mathrm{emb}}=F_{B}+\Omega_{A}^{\mathrm{elst}}+\Omega_{A}^{\mathrm{exch}}+\Omega_{A}^{\mathrm{LM}} \end{array}$$

For the orbitals at stationary conditions, the generalized Brillouin condition for the dimer forces the off-diagonal occupied–virtual block of the embedded Fock matrices to vanish, (*F*_A_^emb^)_α_^ρ^ = 0 and (*F*_B_^emb^)_β_^σ^ = 0. The occupied–occupied and virtual–virtual blocks of effective subsystem Fock matrices are not determined by these conditions, which leaves some freedom in their definition. Here, we have defined the full subsystem effective Fock matrices following the commutator form of the (generalized) Fock matrix (see, e.g., Rowe et al.^76^ or Chapter 10 of ref 77):

24 $${\left(F_{X}^{\mathrm{emb}}\right)}_{k}^{l}=\left\langle \Phi_{X}\right|\left\{x_{l}^{†},\left[H_{X},x_{k}\right]\right\}\left|\Phi_{X}\right\rangle$$

where *X* can be either A or B with *x* ^†^, *x* operators becoming *a* ^†^, *a* or *b* ^†^, *b* appropriately. In our case, *H* is the total dimer operator used in the stationarity analysis, *H* → $\frac{1}{2}\left(\left(H_{A}+H_{B}+V\right)A+A\left(H_{A}+H_{B}+V\right)\right)$ , so eq 24 yields generalized Fock matrices for each monomer consistent with the variational formulations. This construction closely parallels earlier generalized-Fock matrices^78–80^ and aligns with the commutator matrices used in the extended Koopmans’ theorem evaluations of ionization potentials/electron affinities.^81–83^

The detailed expressions for the electrostatic, Pauli exchange, and Landshoff–Murrell potentials will be given in Section 3.

### 2.3 Symmetry-Adapted Relaxation

Equations 21 and 22 introduced in the previous section are among the most important results of the present work, as they define effective potentials that describe the Pauli exclusion (exchange) effects *without* requiring an explicit orthogonalization of the A and B orbital spaces, thereby preventing intermonomer orbital mixing and preserving monomer identity.

Consequently, the dimer variational principle leads to self-consistent equations with embedded Fock matrices **F**_A_^emb^ and **F**_B_^emb^ where all Ω-potentials are evaluated self-consistently with the orbitals of A and B. The method therefore relies on relaxing the orbitals of each subsystem in the external field generated by its partner. We will refer to this approach as the *Symmetry-Adapted Relaxation Theory* (SART), since the subsystem orbitals relax in the symmetry-adapted fields of their counterparts (see Section 3.1 for details of the self-consistent scheme). Crucially, these fields include not only the electrostatic potential, but also the exchange and Landshoff–Murrell potentials; it is precisely the inclusion of these terms that preserves the antisymmetry of the wave function and prevents the overpolarization that plagues the Hartree–Hartree–Fock approach.

While this paper is based on the Hartree–Fock wave functions and Hartree–Fock orbital relaxation, we would like to point out that the variational Ansatz can be chosen in a more complex form. Thus, it is possible to extend the method to, for example, MCSCF, configuration interaction, or any variational quantum chemistry method. The method could be very easily adapted to the DFT case, simply replacing the Hartree–Fock orbitals with the Kohn–Sham ones.

A key requirement—and our primary validation check—is that, for an HF reference, the self-consistent solution of the modified equations reproduces the *supermolecular HF interaction energy*. This limit guarantees that the proposed scheme is consistent with the underlying HF theory while providing a symmetry-adapted, nonorthogonal route to the same result. It should also be stressed that we are able to reproduce that limit without ever calculating the dimer wave function.

## 3 Implementation

The second quantization-based derivation of the SART formulas was performed with our SymPy-based tool for automatic generation of orbital formulas and implementation of tensor contractions adapted to SAPT-like problems.^57,60,84^ The final MO-based spin–orbital expressions are given in Appendix A below. For exchange and LM potentials, we provide formulas specific for occupied-virtual orbital blocks (OV) along with occupied–occupied (OO) and virtual–virtual blocks (VV). Note that all blocks are symmetric and the VO block is identical to OV.

### 3.1 The SART Solver

The SART algorithm is based on the mutual relaxation of the monomers in the presence of the effective partner-induced potentials; the workflow is summarized in the flowchart in Figure 1. To achieve this relaxation in a self-consistent manner, we introduce an iterative cycle comprising consecutive *update* and *relaxation* steps. In each update step, we evaluate/update the SART potentials arising due to partner monomer using orbitals from the previous iteration or the orbitals from isolated monomer calculations in case of initial guess. Once the set of SART potentials is constructed, the self-consistent relaxation steps for the monomers can be performed, allowing monomer relaxation in the presence of the SART potential arising from the partner interactions. When this relaxation is done to self-consistency, we get the *self-consistent* algorithm, but if we instead perform only one iteration of the monomer relaxation step, we instead get the *diagonal* approach. After each set of update and relaxation step(s), we obtain relaxed orbitals within the diagonal or self-consistent schemes for both monomers and the SART interaction energy, *E*_int_^SART^, is evaluated using eq 15. The convergence of the SART update-relaxation steps is determined using an energy change criterion based on the SART interaction energy (eq 15): convergence is achieved when |*E*_int_^SART,[*n*+1]^ – *E*_int_^SART,[*n*]^| < ϵ where ϵ is a suitable energy tolerance on the interaction energy. Note that the convergence is determined directly on the basis of the interaction energy.

**Figure 1:**
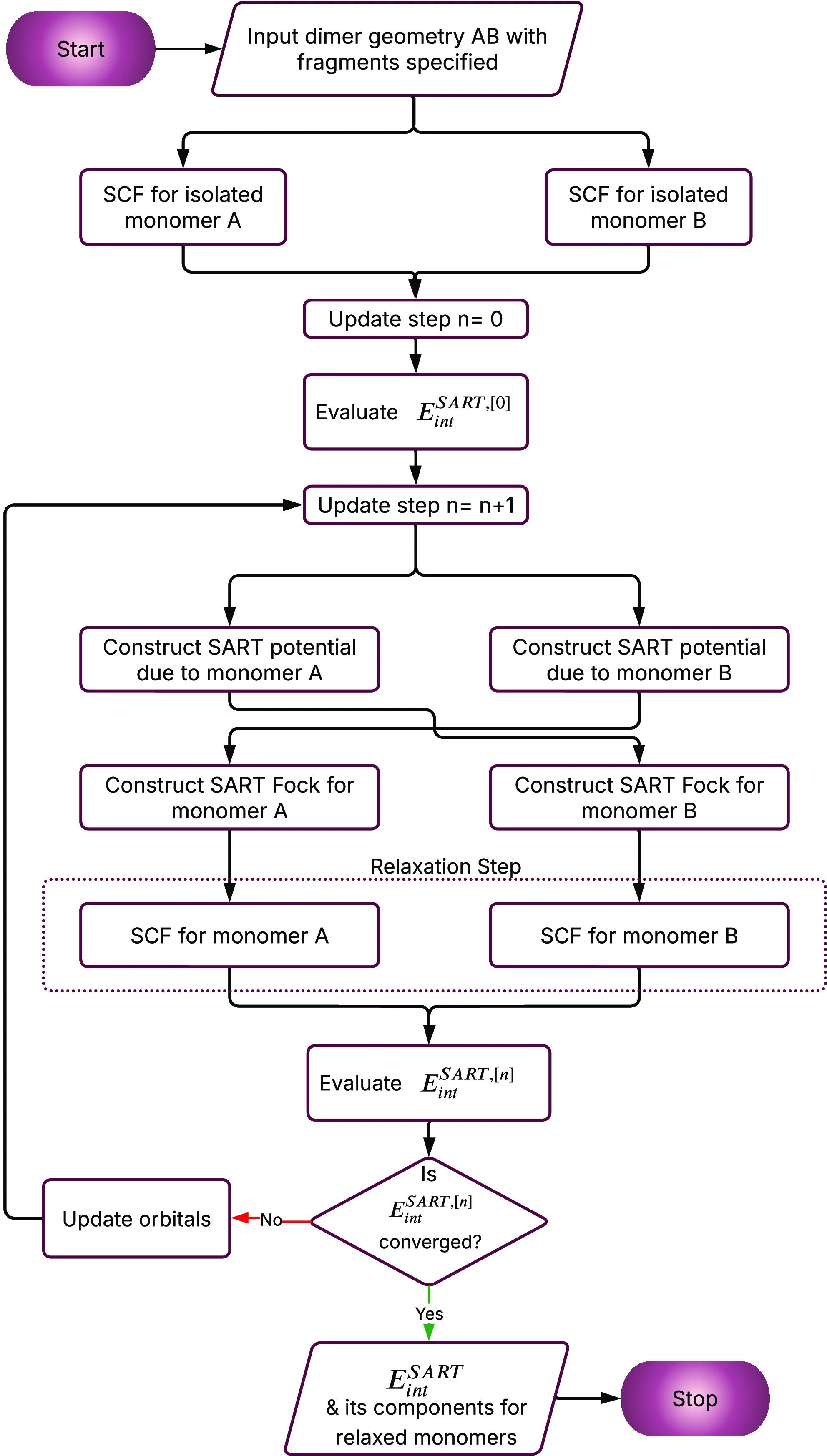
Workflow for the orbital optimization in the Symmetry-Adapted Relaxation Theory (SART).

### 3.2 Numerical Details

The second-quantized (SQ) approach of SART is implemented in the Python language and is integrated with the open-source Psi4 quantum chemistry package^85^ using its Python interface Psi4Numpy,^86^ with the tensor contraction engine opt-einsum.^87^ The SQ approach is limited to the dimer-centered basis set (DCBS): consequently, only such basis sets were used in the present study. All SART and reference calculations were performed using the aug-cc-pVTZ basis set. For benchmarking and comparison, we have evaluated the intermolecular interaction energy using supermolecular Hartree–Fock and SAPT0 with Psi4.^85^ The energy threshold for the calculation is kept at 10^–7^ *E*_*h*_. As evident from our orbital expressions given in the previous section and Appendix A, our implementation adopts a molecular orbital (MO) representation for the closed-shell (spin-summed) SART potential expressions, though an AO-based implementation is planned.

The current implementation of SART is based on the SCF module of Psi4, where the Ω_A_ and Ω_B_ potentials are introduced as external potentials which depend on densities from the previous iterations. The computational cost of evaluating the SART potential within the SQ framework scales as $O\left(N^{4}\right)$ , where, for a given basis type, *N* scales with the number of atoms and is proportional to the number of occupied and virtual orbitals. However, the present MO-based implementation is limited by the AO-to-MO transformation of the standard two-electron repulsion integrals, which scales as $O\left(N^{5}\right)$ .

## 4 Results

The example systems studied in this paper are H_2_O···H_2_O, Li^+^···H_2_O and F^–^···H_2_O. Figure 2 shows the geometry configurations of these systems. For H_2_O···H_2_O, the geometries used are taken from the reference S66 database^88^ of benchmark interaction energies, while the F^–^···H_2_O equilibrium geometry is obtained from the study on the assessment of methods for the polarizable force field development.^89^ For the Li^+^···H_2_O, the geometry is chosen to sample cation–lone-pair interaction. The equilibrium geometries are available in the Supporting Information.

**Figure 2:**
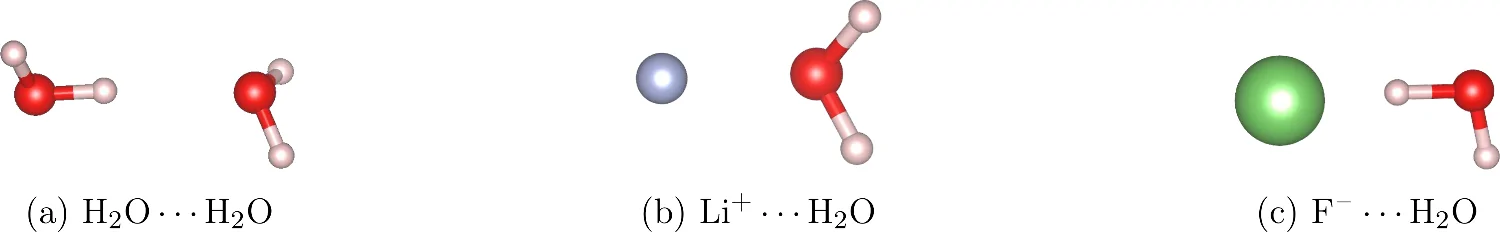
Equilibrium geometries of the test systems.

We have chosen these systems as all three are strongly bound, with relatively short equilibrium separations. These three systems all pose a challenge for SAPT as all are strongly bound and have substantial contributions from induction effects beyond second order in the interaction operator. Additionally, H_2_O···H_2_O and F^–^···H_2_O are known to exhibit substantial charge delocalization^89,90^ and, due to the short equilibrium separations, intermolecular exchange energies must be calculated with the exact antisymmetrization: the single exchange approximation is known to be inadequate and can severely underestimate the exchange energy in such a case.^75,89^ Consequently, these complexes would pose a fundamental problem for any embedding scheme that did not simultaneously account for the full exchange effects and for intermolecular charge delocalization (charge transfer).

Throughout this section, distances are reported as the reduced separations in units of *R*_eq_. Table 2 (shown later in this work) reports SART­[HF] and component energies alongside SAPT0 at the equilibrium geometries (*R*/*R*_eq_ = 1.0) while Table S1 in the Supporting Information presents the same decomposition at *R*/*R*_eq_ = 2.0 to shed some light on the behavior in the asymptotic regime.

### 4.1 SART Interaction Energy and Convergence

The primary test for SART is that it is able to reproduce the supermolecular Hartree–Fock interaction energy, *E*_int_^HF^, which contains the effects of infinite-order induction, without intramolecular electron correlation but with proper exchange quenching. We can regard these calculations on dimers as the simplest case of embedding: each of the two molecules is *embedded* in the potential arising from the other, and the SART iterations ensure that the embedding is self-consistent. The calculations we report are difficult in two important ways. First, we use the dimer basis set which is the worst-case basis set for standard electrostatic embedding methods due to the catastrophic overpolarization that would usually result as a consequence of having basis functions on the nuclei of the embedding environment atoms. Second, we report results for strongly interacting systems, some high on the repulsive wall, where exchange-repulsion and charge delocalization effects are both large. Consequently, the ability of the SART formalism to correctly recover the supermolecular Hartree–Fock interaction energy under these conditions will validate the methodology.

In the upper panels of Figures 3, 4, and 5, we see interaction energies as well as SART components calculated for the H_2_O···H_2_O, Li^+^···H_2_O, and F^–^···H_2_O systems, as a function of the separation between the molecules. In all cases, we see that even in the repulsive geometries, it is impossible to distinguish between the supermolecular interaction energy, *E*_int_^HF^, and that from converged SART­[HF], *E*_int_^SART[HF]^. In Tables 1, 2, and Table S1 in the Supporting Information, we report numerical data for the interaction energies of these dimers at short-range (on or near the repulsive wall), equilibrium, and long-range. For all three systems, at all three sets of separations, the difference in *E*_int_^HF^ and *E*_int_^SART[HF]^ is only of the order 0.001% for interaction energies that range from –49 mE_h_ (−31 kcal/mol) to 576 mE_h_ (361 kcal/mol). At the shortest separations (Table 1), the exchange energies *E*_exch_ range from 52 mE_h_ (33 kcal/mol) to 1224 mE_h_ (768 kcal/mol), that is, the effects of intermolecular overlap are large and any method that neglects or only crudely approximates exchange effects might be expected to fail. Indeed, we will investigate approximations to the exchange and other terms in Section 4.3.

**Figure 3:**
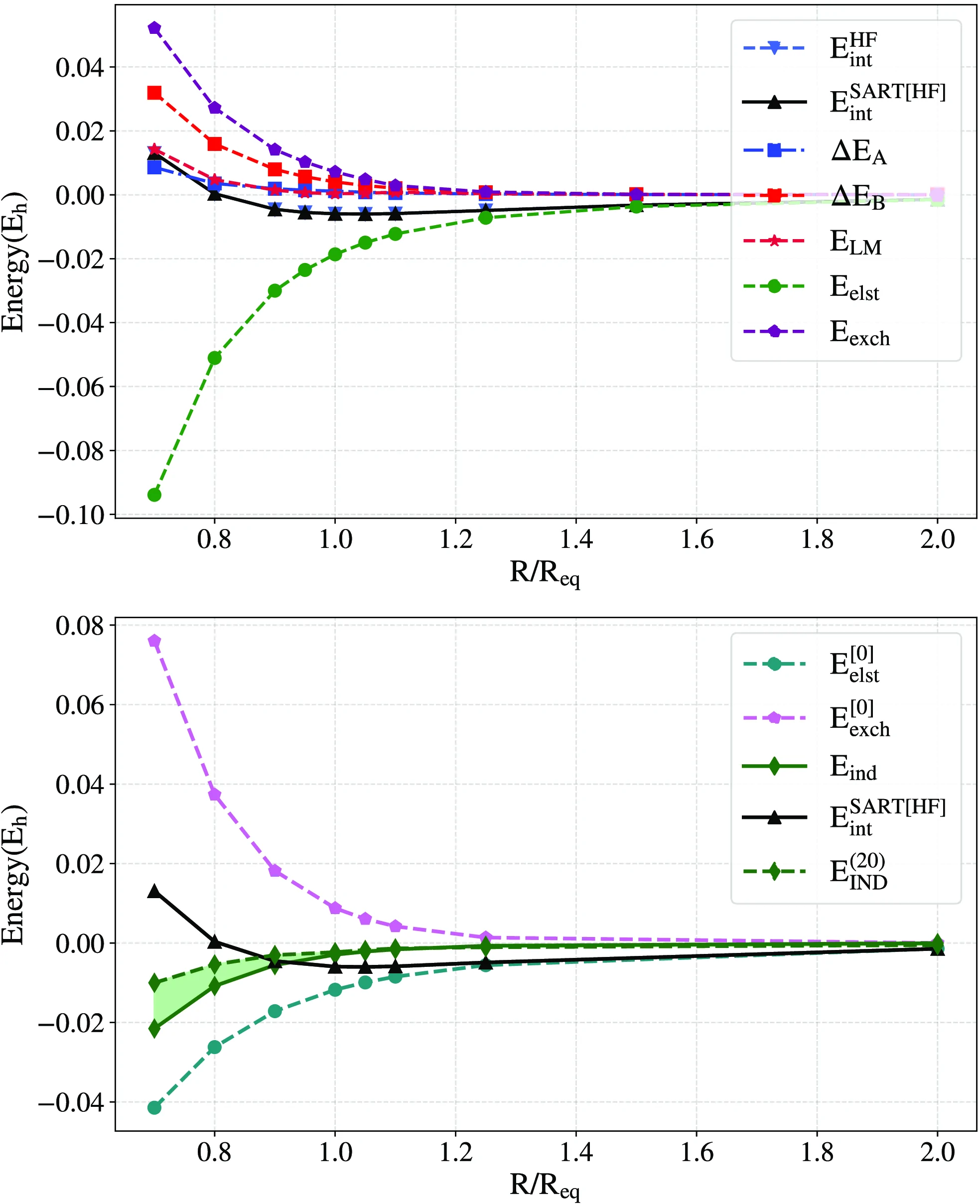
Interaction energies computed with SART for the water dimer along the intermolecular H···O hydrogen-bond stretch of the equilibrium geometry complex (*R*/*R*_eq_ = 1 corresponds to the minimum). The equilibrium separation *R*_eq_ (H···O) = 2.011 Å, where *E*_int_^HF^ = −5.937 mE_h_. The SART convergence is achieved using the fully relaxed scheme. The upper panel shows the primary SART components and the lower panel shows the physical SART components as well as a comparison with the SAPT0 *E*_IND_^(20)^ values. The shaded area highlights the difference between *E*_IND_^(20)^ and *E*_ind_.

**Figure 4:**
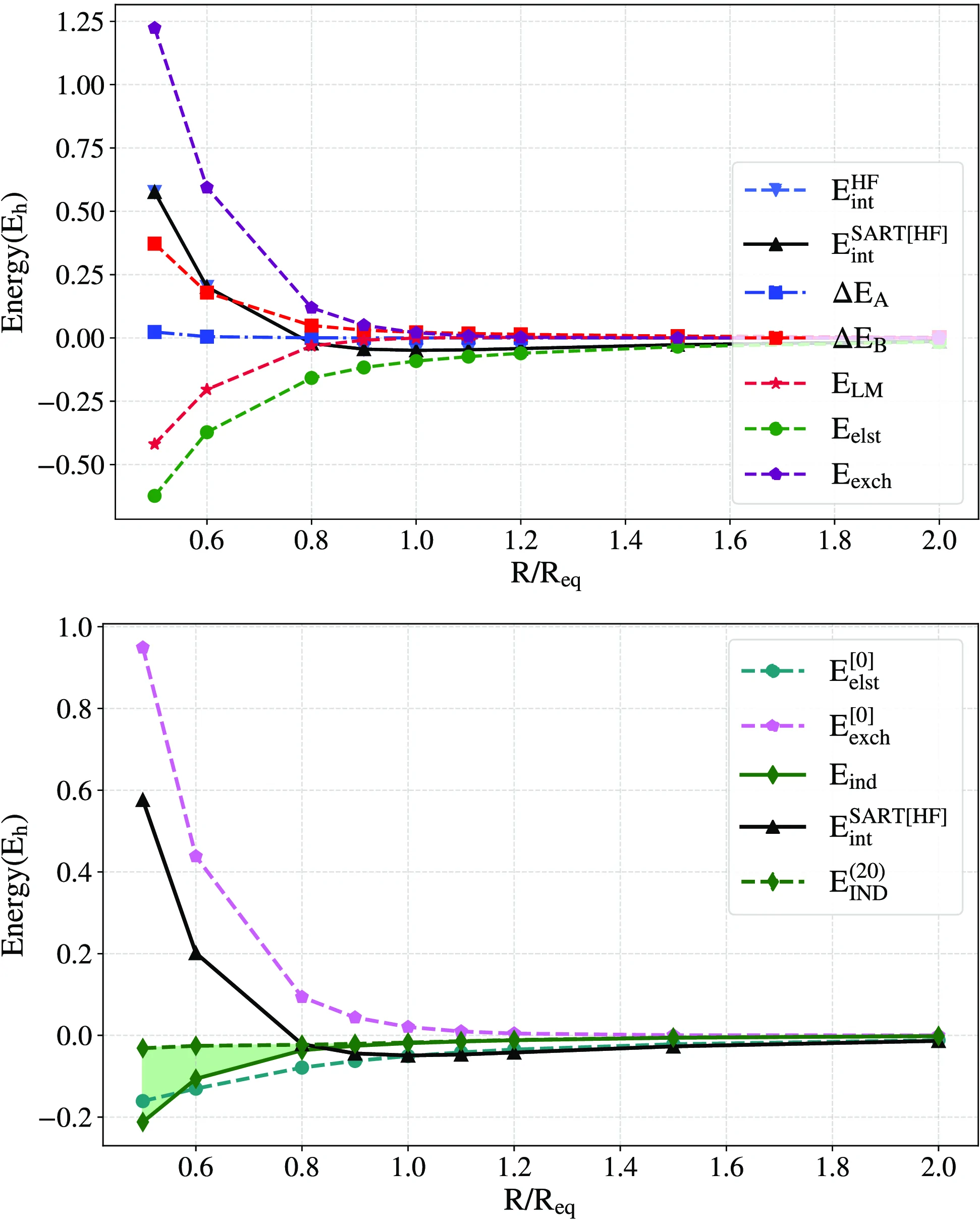
Interaction energies computed with SART for the Li^+^···H_2_O dimer along the Li···O stretch of the equilibrium geometry complex (*R*/*R*_eq_ = 1 corresponds to the minimum). The equilibrium separation, *R*_eq_(Li···O) = 1.87 Å (*E*_int_^HF^ = −49.20 mE_h_). The SART convergence is achieved using the fully relaxed scheme. The upper panel shows the primary SART components and the lower panel shows the physical SART components as well as a comparison with the SAPT0 *E*_IND_^(20)^ values. The shaded area highlights the difference between *E*_IND_^(20)^ and *E*_ind_.

**Figure 5:**
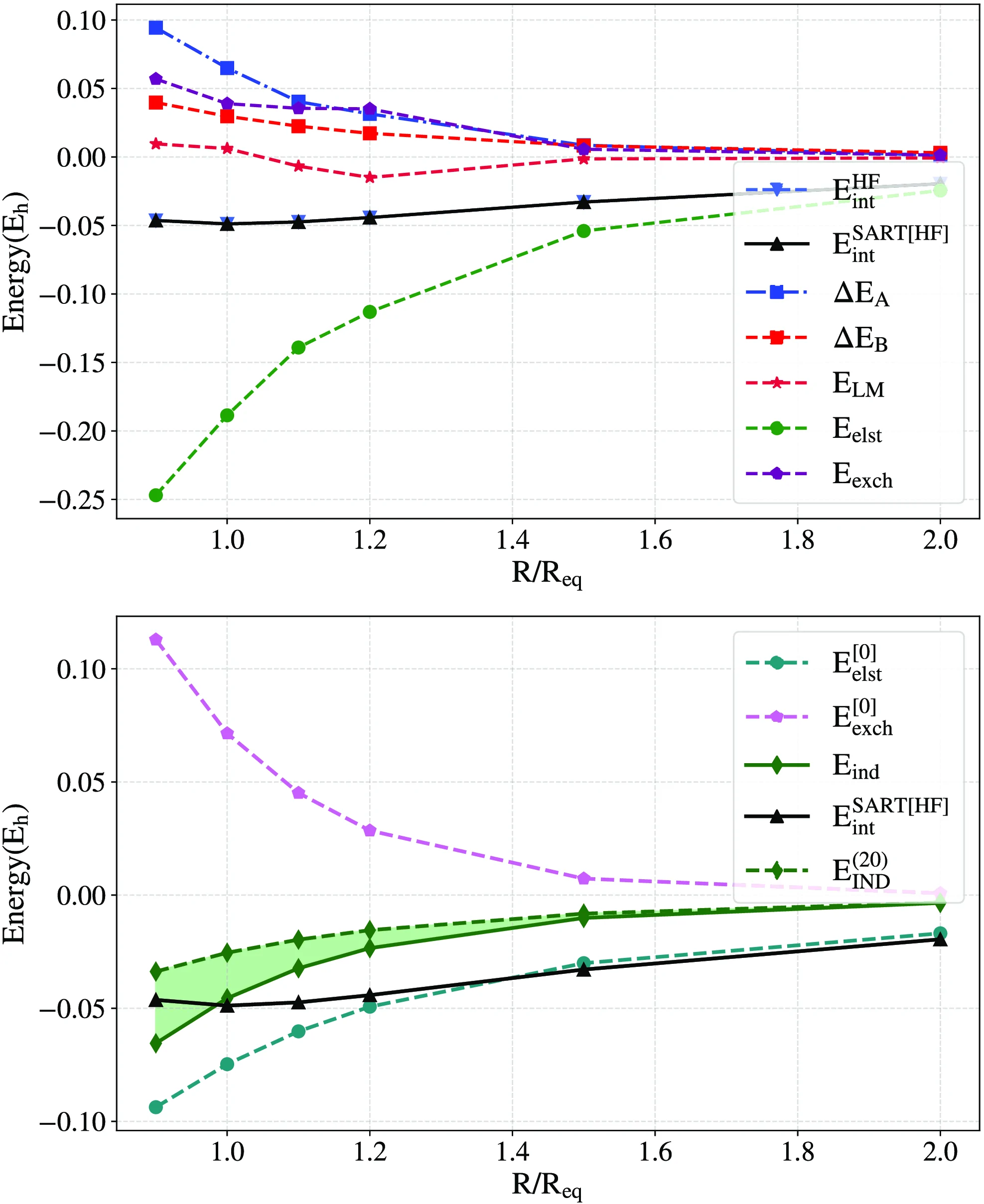
Interaction energies computed with SART for the F^–^···H_2_O system along the F···H stretch of the equilibrium geometry complex (*R*/*R*_eq_ = 1 corresponds to the minimum). The equilibrium separation is *R*_eq_(F···H) = 1.374 Å (*E*_int_^HF^ = −48.847 mE_h_). The SART convergence is achieved using the diagonally relaxed scheme. The upper panel shows the primary SART components and the lower panel shows the physical SART components as well as a comparison with the SAPT0 *E*_IND_^(20)^ values. The shaded area highlights the difference between *E*_IND_^(20)^ and *E*_ind_.

**Table 1:** SART­[HF] Energy Contributions in Comparison to the SAPT0 Energies for H_2_O···H_2_O, Li^+^···H_2_O, and F^–^···H_2_O at *R*/*R*_eq_ = 0.7, 0.5, and 0.9, Respectively^a^

System	H_2_O···H_2_O	Li^+^···H_2_O	F^–^···H_2_O
Distance (Å)	1.408	0.935	1.237
			
SAPT0 interaction energy components:			
*E*_elst_^(10)^	–41.446	–160.838	–93.728
*E*_exch_^(10)^	76.035	949.107	112.910
*E*_IND_^(20)^	–10.012	–31.318	–33.784
*E*_ind,resp_^(20)^	–36.375	–402.277	–76.608
*E*_exch–ind,resp_^(20)^	26.363	370.958	42.823
			
SART primary components:			
Δ*E*_A_	8.588	23.338	94.360
Δ*E*_B_	31.927	372.078	39.741
*E*_LM_	14.251	–419.153	9.573
*E*_L,A_	–1.376	–91.603	50.120
*E*_M,A_	1.103	15.043	0.802
*E*_L,B_	13.749	–361.176	–43.416
*E*_M,B_	0.775	18.583	2.068
*E*_elst_	–93.894	–623.625	–246.930
*E*_exch_	52.189	1223.609	56.939
			
SART physical components:			
*E*_elst_^[0]^	–41.446	–160.838	–93.728
*E*_exch_^[0]^	76.035	949.107	112.910
*E*_ind_	–21.529	–212.020	–65.499
*E*_def_	54.766	–23.736	143.674
*E*_def,A_	8.314	–53.222	145.281
*E*_def,B_	46.451	29.486	–1.607
*E*_rel_	–76.295	–188.284	–209.173
Δ*E*_elst_	–52.448	–462.787	–153.203
Δ*E*_exch_	–23.847	274.503	–55.970
			
*E*_int_^SAPT0^ (no disp)	24.577	756.950	–14.602
*E*_int_^HF^	13.060	576.248	–46.318
*E*_int_^SART[HF]^	13.060	576.248	–46.317
Rel. error (%)	0.000 993	0.000 017	0.002 097

a All energy values are in millihartree (mE_h_). The distances are between H···O, Li^+^···O, and F^–^···H, respectively.

**Table 2:** SART­[HF] Energy Contributions in Comparison to the SAPT0 Energies for H_2_O···H_2_O, Li^+^···H_2_O, and F^–^···H_2_O at Equilibrium Geometries (*R*/*R*_eq_ = 1.0)^a^

System	H_2_O···H_2_O	Li^+^···H_2_O	F^–^···H_2_O
Distance (Å)	2.011	1.870	1.375
			
SAPT0 interaction energy components:			
*E*_elst_^(10)^	–11.763	–50.551	–74.719
*E*_exch_^(10)^	8.757	20.507	71.464
*E*_IND_^(20)^	–1.764	–17.458	–25.456
*E*_ind,resp_^(20)^	–3.769	–33.674	–49.854
*E*_exch–ind,resp_^(20)^	2.005	16.216	24.398
			
SART primary components:			
Δ*E*_A_	1.141	0.139	64.906
Δ*E*_B_	4.037	22.287	29.713
*E*_LM_	0.342	–0.613	6.180
*E*_L,A_	–2.290	–0.175	39.369
*E*_M,A_	0.037	0.024	0.501
*E*_L,B_	2.562	–0.492	–34.847
*E*_M,B_	0.034	0.031	1.156
*E*_elst_	–18.628	–91.338	–188.564
*E*_exch_	7.171	20.325	38.920
			
SART physical components:			
*E*_elst_^[0]^	–11.763	–50.551	–74.719
*E*_exch_^[0]^	8.757	20.507	71.464
*E*_ind_	–2.930	–19.155	–45.591
*E*_def_	5.520	21.813	100.799
*E*_def,A_	–1.113	–0.012	104.777
*E*_def,B_	6.633	21.825	–3.978
*E*_rel_	–8.450	–40.969	–146.390
Δ*E*_elst_	–6.864	–40.787	–113.846
Δ*E*_exch_	–1.586	–0.182	–32.544
			
*E*_int_^SAPT0^ (no disp)	–4.770	–47.502	–28.711
*E*_int_^HF^	–5.937	–49.200	–48.847
*E*_int_^SART[HF]^	–5.937	–49.199	–48.845
Rel. error (%)	0.000 936	0.000 106	0.002 647

a All energy values are given in millihartree (mEh). The distances are between H···O, Li^+^···O, and F^–^···H, respectively. The SAPT0 energy corrections, SART­[HF] primary energy components, and the physical energy components are shown in consecutive row blocks. The splitting of energies by the sub-components and individual monomers is also highlighted.

These results demonstrate that the SART approach does correctly describe the intermolecular embedding potentials, and that we are able to self-consistently include nondispersion intermonomer interactions. Even though all we have shown so far is that we can recover the supermolecular Hartree–Fock interaction energy using the SART­[HF] approach, there is one essential difference in the methods: within the SART formalism we recover the intermolecular interaction energy directly, without ever computing the supermolecular wave function or the supermolecular energy.

In Figure 6 we visualize the convergence of the SART­[HF] components for the H_2_O ··· H_2_O, Li^+^ ··· H_2_O and F^–^ ··· H_2_O systems at their equilibrium geometries as a function of the iteration (update) number. Usually we converge energies to 10^–7^ to 10^–9^ E_h_, as in the supermolecular approach the monomer energies need to be very precisely determined for the interaction energy to be computed as the difference of dimer and monomer energies. In the case of SART, we do not take energy differences, but instead compute the SART­[HF] interaction energy as a sum of terms as shown in eq 15. Consequently, while we will demonstrate convergence of E_int_^SART[HF]^ with a tight threshold of 10^–7^ E_h_, a more realistic convergence threshold might be larger than 10^–5^ E_h_ (0.01 kcal/mol). Both thresholds, tight and realistic, are indicated in Figure 6. Among these three test systems, the convergence of SART is obtained relatively faster in case of H_2_O ··· H_2_O (update 4) and Li^+^ ··· H_2_O (update 3), while for the F^–^ ··· H_2_O (update 21) system convergence proved to be relatively challenging and was only obtained using the diagonal scheme (see Sec. [Sec sec3.1]).

**Figure 6:**
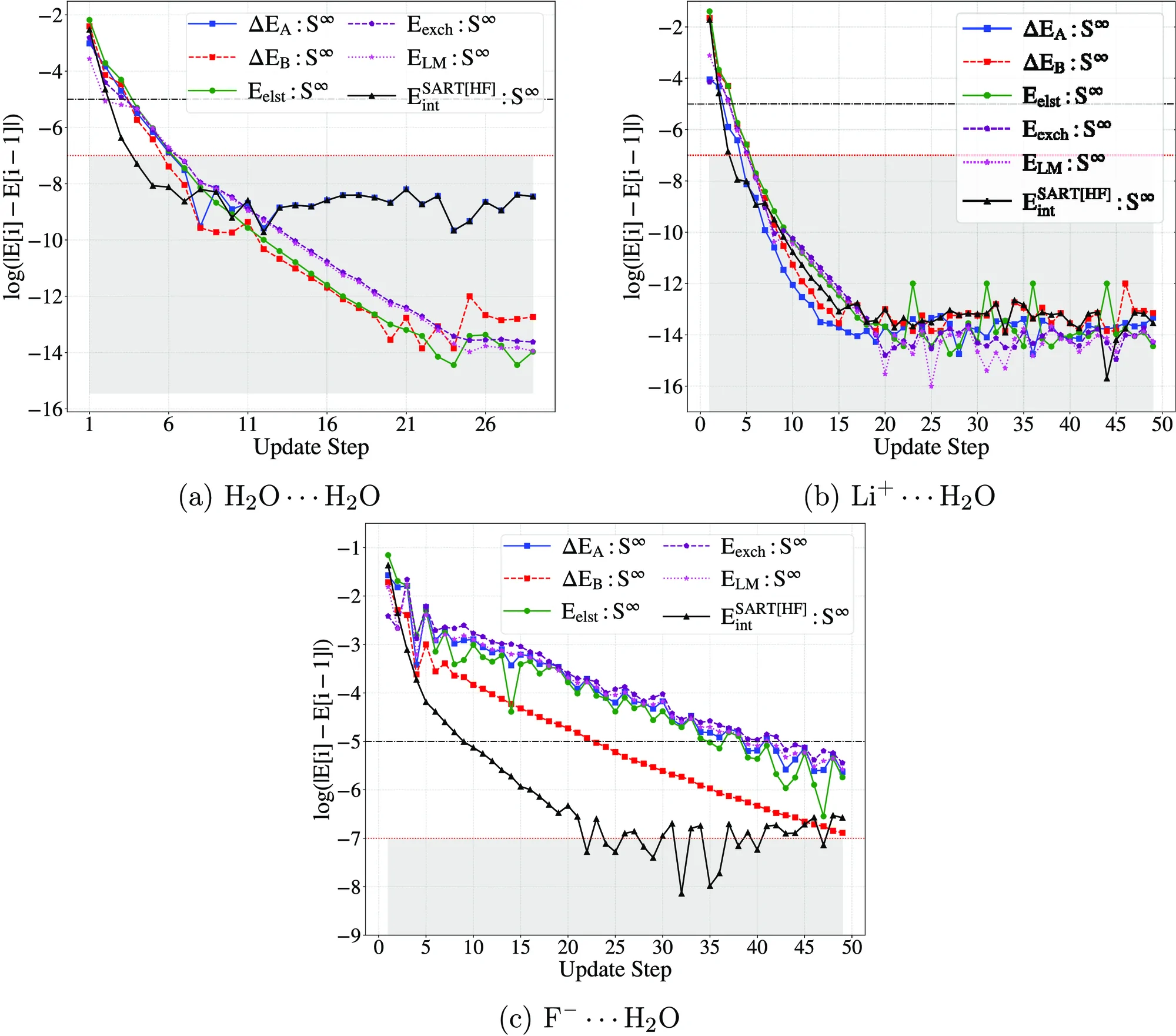
Convergence pattern of *E*_int_^SART[HF]^ and its primary components for H_2_O···H_2_O, Li^+^···H_2_O, F^–^···H_2_O with the full Ω^exch^ and Ω^LM^ potentials at equilibrium geometry. In each plot, the logarithms of the absolute differences between consecutive energy values are plotted. The black horizontal line represents the reference accuracy of interaction energy, 10^–5^ *E*_h_ and the red dotted line represents the convergence threshold, 10^–7^ *E*_h_ for the SART scheme. The convergence for *E*_int_^SART[HF]^ is achieved using self-consistent relaxation after iteration 4, 3 in Figures 6a, 6b for H_2_O···H_2_O and Li^+^···H_2_O respectively. For F^–^···H_2_O in Figure 6c, the realistic convergence of 10^–5^ *E*_h_ is achieved after iteration 9 using the diagonally relaxed scheme.

In Figures S1, S2, and S3 in the Supporting Information, we show the convergence patterns for these systems at their short-range and long-range geometries. The convergence is fastest at long-range (*R*/*R*_eq_ = 2.0): from Figure S1b for the H_2_O···H_2_O complex, we see convergence in one iteration for the realistic threshold and three for the tight threshold. We have not done a detailed study of SART convergence patterns, but we can observe from Figure S1b that the SART interaction energy converges in two exponential stages until iteration 15, after which no further meaningful change occurs. Even at the shortest separation of the H_2_O···H_2_O (see Figure S1a), *E*_int_^SART[HF]^ converges to the tight threshold in less than 5 iterations. However, the individual SART energy components may take more iterations to converge to the same tolerance.

The F^–^···H_2_O system proved to be somewhat more challenging for the present implementation of the SART iterative procedure. While we were able to achieve convergence to the tight threshold at long-range (Figure S3b), at short-range (Figure S3a), as at equilibrium (Figure 6c), it is not clear if the present algorithm is able to converge to the tight threshold of 10^–7^ E_h_. This may have to do with the significantly stronger binding seen in F^–^···H_2_O and the possibly large charge reorganization associated with the unusually strong intermolecular bond, which has a binding energy of −48.85 mE_h_.

For the cationic system Li^+^···H_2_O at long- and short-range (Figure S2) convergence is fastest: only 2 update steps are needed at long-range (*R*/*R*_eq_ = 2.0), and 20 updates at very short-range (*R*/*R*_eq_ = 0.5). This is perhaps not too surprising as the higher-order induction in this complex becomes important only at very short intermolecular separations (the lower panel in Figure 4), perhaps due to the small polarizability of the cationic species.

### 4.2 SART Interaction Energy Components

We now focus on understanding the SART interaction energy components and how we can associate them with terms from SAPT. At first glance, the SAPT0 and SART interaction energy components shown in Tables 1, 2, and Table S1 in the Supporting Information do not seem related. There is a point of similarity—at least in name—in the electrostatic and exchange terms, but even these differ substantially between the methods. For example, on the repulsive wall (Table 1), for F^–^···H_2_O, *E*_elst_^(10)^ = −93.73 mE_h_, but the SART electrostatic energy *E*_elst_ = −246.93 mE_h_ is more than twice larger in magnitude. Likewise, for the same system, the SAPT0 exchange *E*_exch_^(10)^ = 112.91 mE_h_ is more than twice as large as the corresponding SART exchange *E*_exch_ = 56.94 mE_h_. Furthermore, the SART components contain terms of zeroth leading order in the interaction operator and therefore are more naturally associated with monomer energy changes. These do not seem to be readily associated with an intermolecular interaction energy component from SAPT0. However, as we now demonstrate, there is a strong and insightful relation between the two.

The first point of association between the two methods, SAPT0 and SART­[HF], is found at the zeroth SART update step (*n* = 0 in Figure 1): here, the SART interaction energy in eq 15 is evaluated with unrelaxed monomer wave functions just as is done in the first-order SAPT0 energy expressions. Here, only the electrostatic and exchange terms are nonzero, and these are unsurprisingly equal to the corresponding terms from SAPT0. If we denote by *E*_elst_^[0]^ and *E*_exch_^[0]^ the SART­[HF] electrostatic and exchange energies at update step *n* = 0, then we have *E*_elst_^[0]^ = *E*_elst_^(10)^ and *E*_exch_^[0]^ = *E*_exch_^(10)^. Having established this, it then follows that the remainder of the SART interaction energy must be associated with relaxation effects, that is, with the induction energy. We now see how this can be understood.

All of the SART interaction energy beyond the first update step is associated with the self-consistent relaxation process, and includes two kinds of terms: an attractive intermolecular relaxation energy, *E*_rel_, and a deformation energy penalty, *E*_def_. The intermolecular relaxation energy is defined as the sum of the electrostatic and exchange relaxation terms as

25 $$E_{\mathrm{rel}}\overset{\mathrm{def}}{\equiv }\Delta E_{\mathrm{elst}}+\Delta E_{\mathrm{exch}}$$

where

$$\begin{array}{l} \Delta E_{\mathrm{elst}}\overset{\mathrm{def}}{\equiv }E_{\mathrm{elst}}-E_{\mathrm{elst}}^{\left[0\right]}=E_{\mathrm{elst}}-E_{\mathrm{elst}}^{\left(10\right)},\mathrm{and}\\ \Delta E_{\mathrm{exch}}\overset{\mathrm{def}}{\equiv }E_{\mathrm{exch}}-E_{\mathrm{exch}}^{\left[0\right]}=E_{\mathrm{exch}}-E_{\mathrm{exch}}^{\left(10\right)} \end{array}$$

These definitions naturally identify the energy of stabilization from the relaxation process as arising from the intermolecular electrostatic and exchange interactions that occur on monomer wave function relaxation. But there is an energetic price to pay for this relaxation from the free-monomer wave functions and this is the deformation energy penalty.

The deformation energy contribution from each monomer is defined as the energy difference between the monomer energy at self-consistency, in the complex, and the free (or reference) monomer energy. Using eqs 6, 11, and 12, we obtain the following for monomer A:

26 Edef,A≡def⟨ΦAΦB|HAA|ΦAΦB⟩L−EA=(⟨ΦAΦB|HA|ΦAΦB⟩−EA)+⟨ΦAΦB|HAP|ΦAΦB⟩L=ΔEA+ELM,A

An analogous definition holds for monomer B. The presence of the LM term in eq 26 is why we have termed Δ*E*_A_ as the *apparent* deformation: Δ*E*_A_ is computed without the intermolecular antisymmetrizer, and therefore is not the true expectation value of *H*_A_ in the nonorthogonal space in which we operate. The total deformation energy can be written as the sum of the deformation energies of both monomers:

27 $$E_{\mathrm{def}}\overset{\mathrm{def}}{\equiv }E_{\mathrm{def},A}+E_{\mathrm{def},B}$$

Finally, we combine the total deformation energy and relaxation energy to define the induction energy in SART as follows:

28 $$E_{\mathrm{ind}}\overset{\mathrm{def}}{\equiv }E_{\mathrm{def}}+E_{\mathrm{rel}}$$

This is the induction energy at convergence, and so *E*_ind_ can be regarded as the *infinite-order* induction in the SAPT sense. This treatment of the induction energy closely mirrors that of the classical polarization model (see, for example, Chapter 9 in the work by Stone^91^) which also includes an explicit deformation term that partially quenches the energy of stabilization from relaxation. One essential difference is that classical polarization models are usually constructed assuming a linear response of the change in a multipole moment with field strength. This leads to the deformation term being exactly half the magnitude of the relaxation term (see, for example, Chapter 5 in the work by Israelachvili^92^), but this is only approximately the case in SART, as all orders of response are included implicitly in the relaxation process.

The SART interaction energy *E*_int_^SART^ can then be expressed in terms of the SART physical energy components as

29 $$E_{\mathrm{int}}^{\mathrm{SART}}=E_{\mathrm{elst}}^{\left[0\right]}+E_{\mathrm{exch}}^{\left[0\right]}+E_{\mathrm{ind}}$$

For SART­[HF], the first two terms are identical to *E*_elst_^(10)^ and *E*_exch_^(10)^ from SAPT0, and the *E*_ind_ term is the induction energy, up to infinite order in the interaction operator, obtained from the relaxed monomers.

Note that for SART based on Hartree–Fock, this composition of the interaction energy closely mirrors the familiar SAPT0 picture if the δ_int_^HF^ term is used to estimate the higher than second-order induction contributions not included in SAPT0. However, for SART based on KS-DFT or correlated monomers, the mapping is no longer trivial, but further work is needed to understand what we recover from SART in these cases. Nevertheless, even for SART­[HF], there is one essential point of difference from SAPT0 with δ_int_^HF^ included: the SART formalism is able to recover the δ_int_^HF^ contribution without ever calculating the supermolecular wave function or energy.

The SART­[HF] physical energy components for the H_2_O···H_2_O, Li^+^···H_2_O, and F^–^···H_2_O systems are presented in Tables 1, 2 and Table S1 (see the Supporting Information), and are visualized as a function of intermolecular separation in the lower panels in Figures 3, 4, and 5.

For the water dimer, we compare the SART induction energy, E_ind_, with the second-order SAPT0 induction and exchange-induction, summed into *E*_IND_^(20)^ (Figure 3). At equilibrium (Table 2), *E*_IND_^(20)^ = −1.764 mE_h_, whereas the SART induction *E*_ind_ = −2.930 mE_h_ is much larger in magnitude, with *E*_ind_ accounting for 49% of the total interaction, *E*_int_^SART^ = −5.937 mE_h_. As the intermolecular separation decreases, the underestimation inherent to *E*_IND_^(20)^ becomes increasingly severe, making induction evaluated with the relaxed SART orbitals an essential improvement. At the repulsive geometry (*R*/*R*_eq_ = 0.7, in Table 1) we see that the SART induction *E*_ind_ is more than twice as large as *E*_IND_^(20)^. From Figure 3 we see that, to leading order, *E*_ind_ and *E*_IND_^(20)^ share the same asymptotics. The shaded region between *E*_ind_ and *E*_IND_^(20)^ in the figure denotes the contributions beyond second order; at the HF level, this is precisely what is usually computed using the δ_int_^HF^ term. As described above, the induction energy from SART is expressed as the sum of a deformation term and relaxation term, which for linear response will be in the absolute ratio 1/2. At long-range (*R*/*R*_eq_ = 2.0), from Table S1 (see the Supporting Information), we see that the ratio |*E*_def_/*E*_rel_| = 0.53 which is very close to the linear-response value, but at equilibrium, this ratio is 0.65 and at short-range (*R*/*R*_eq_ = 0.7), it is even larger at 0.72, indicating significant contributions from nonlinear response terms. This is consistent with the increasing importance of the higher-order induction effects at short range.

For the more strongly bound F^–^···H_2_O complex, the pattern is similar to that in the water dimer. Here, we have been unable to sample far into the repulsive wall with the present algorithm, but at equilibrium, there is already a considerable amount of induction effects from contributions beyond second order, as can be seen in the lower panel in Figure 5. It is of interest that the SART physical components are more systematic as a function of *R*/*R*_eq_ than the SART energy components seen in the upper panel of that figure: while *E*_elst_, Δ*E*_A_, and Δ*E*_B_ are monotonic with separation, that is not the case for *E*_exch_ and *E*_LM_, which seem to be correlated in their behavior. We are as yet unsure why this is the case. The ratio |*E*_def_/*E*_rel_| = 0.51 at long range and 0.69 at equilibrium, demonstrating yet again the importance of nonlinear response effects as higher-order induction effects become important.

For the Li^+^···H_2_O complex (Figure 4), the higher-order induction effects are appreciable only on the repulsive wall. At long-range and equilibrium |*E*_def_/*E*_rel_| = 0.53, indicating that we are in the linear-response regime, but at short range (*R*/*R*_eq_ = 0.7), this ratio drops to 0.13 with the *E*_LM_ term quenching the apparent deformation energies from the two monomers. As with F^–^···H_2_O, this may be because of a coupling of the *E*_exch_ and *E*_LM_ terms, and this needs more investigation. We emphasize that, despite this, the total interaction energies remain in excellent agreement with the supermolecular Hartree–Fock ones, and the SART induction, *E*_ind_, remains well-behaved at all separations.

### 4.3 SART Approximations: Importance of the LM Term and the Failure of Expansions in Overlap

In Section 4.1, we have discussed the convergence of SART with no approximations made in the exchange and LM terms. Here, we pose two questions: how important are the LM terms for SART, and what are the consequences for SART if we approximate the LM and exchange terms using expansions in the overlap matrix (*S*)?

Consider the latter question first: expansions in the overlap matrix have been a popular way of deriving approximate SAPT expressions for exchange energies. Indeed, second-order exchange-induction and exchange-dispersion energies were originally derived in the *S* ^2^ approximation and only relatively recently have second-order exchange terms been derived^27,75^ without the expansion in *S*. In a study of systems with substantial induction binding, Naseem-Khan et al.^89^ have shown that the *S* ^2^ approximation in second-order exchange-induction is catastrophic in pure SAPT methods, which may not show any minimum in the interaction energy due to the underestimation of the exchange energies as a result of this approximation. Also recently, Waldrop and Patkowski have shown that the *S* ^2^ approximation leads to even larger errors in the third-order exchange-induction energy,^24^ thus establishing a trend^75^ of increasingly larger underestimation of the exchange in the *S* ^2^ approximation the higher the order in the interaction operator. Since the induction energy in converged SART is effectively of infinite order in the interaction operator, it should come as no surprise that any truncated expansion in powers of the overlap matrix causes the SART iterations to fail catastrophically due to the violations of wave function antisymmetry that the exchange and LM potentials are meant to impose.

In Figure 7, we display the SART­[HF] interaction energies for the H_2_O···H_2_O complex at three separations (*R*/*R*_eq_ = 0.7, 1.0, and 2.0) in two approximations: one with both the exchange and LM energies and potentials truncated to the *S* ^2^ terms, and the second with the milder *S* ^4^ approximation used in the LM term only (the exchange term remains unapproximated). At long range (*R*/*R*_eq_ = 2.0), where overlap effects are negligible, both kinds of approximations lead to converged SART­[HF] interaction energies. However, the convergence picture is much different at shorter separations where overlap effects can no longer be neglected or approximated. At the dimer equilibrium, the *S* ^2^ approximation leads to an apparent convergence (on the scale of the figure) in *E*_int_^SART[HF]^ until iteration 9, after which the energy increases to unphysical values, but the milder *S* ^4^ approximation in the LM term only does appear to result in meaningful interaction energies. However, at short range (*R*/*R*_eq_ = 0.7), neither approximation leads to convergence: now the *S* ^2^ approximation diverges from the start, and while the *S* ^4^ approximation in the LM term leads to quasi convergence until iteration 14, the divergence sets in afterward.

**Figure 7:**
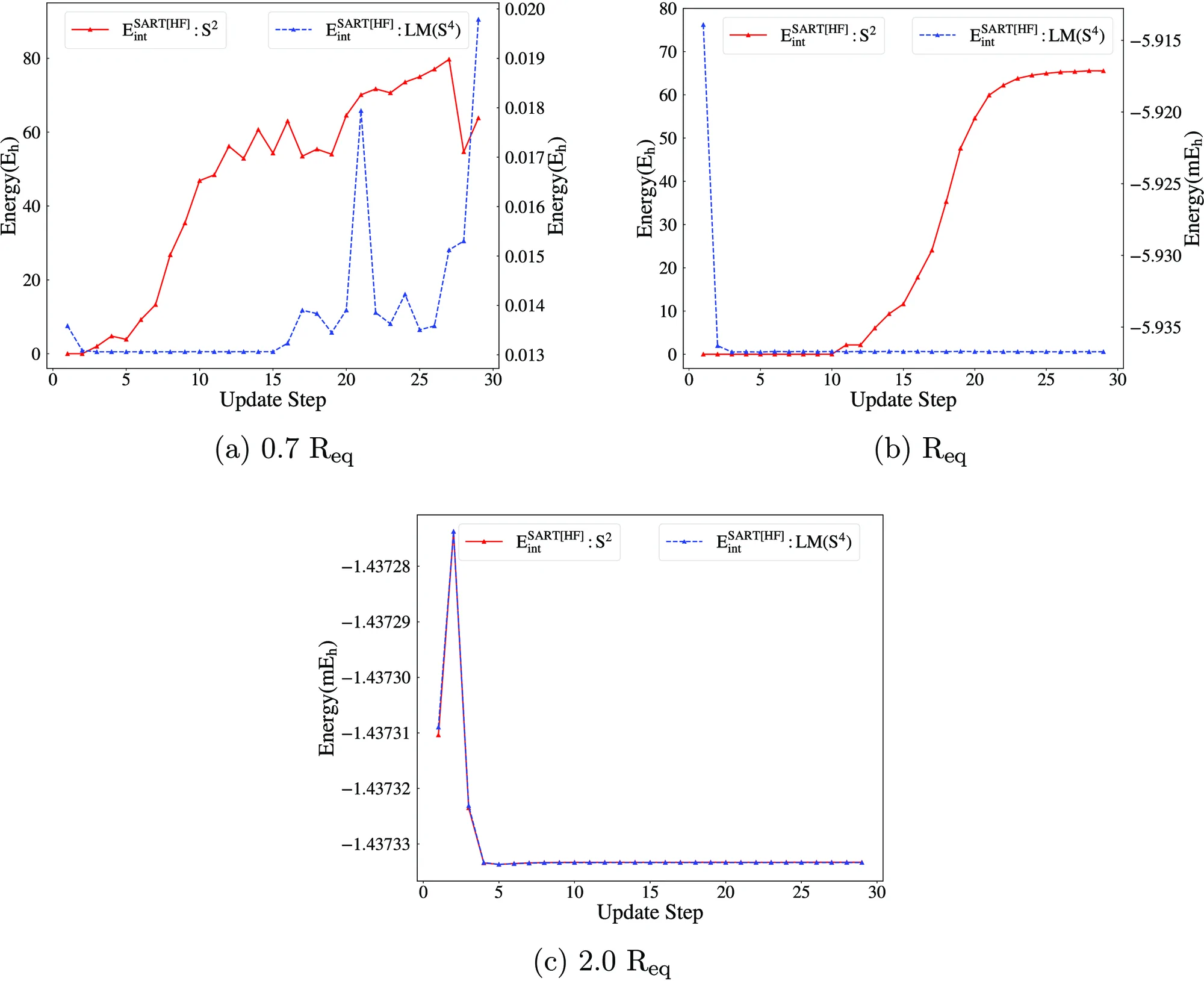
Convergence pattern of the *E*_int_^SART[HF]^ for H_2_O···H_2_O at *R*/*R*_eq_ = 0.7, 1.0, and 2.0, respectively. The update steps, excluding the very first or zeroth one, are shown in the plots. The *E*_int_^SART[HF]^ data for the *S* ^2^ and *S* ^4^ approximation of the SART potentials are plotted using the left and right axes, respectively, except for panel (c), where a common energy scale is used. Here, *S* ^2^ denotes the SART potentials (Ω^exch^, Ω^LM^) evaluated within the *S* ^2^ approximation, and *S* ^4^ denotes Ω^LM^ evaluated with the *S* ^4^ approximation while Ω^exch^ is not approximated.

In Figure 8, we show similar data for SART without the LM terms in the potential as well as the energy: the results are catastrophic at all separations, with a slow initial divergence followed by more rapid, and often erratic, changes in the interaction energy with subsequent iterations. This demonstrates the importance of the LM term for enforcing the antisymmetry of the wave function when nonorthogonal orbitals are used.

**Figure 8:**
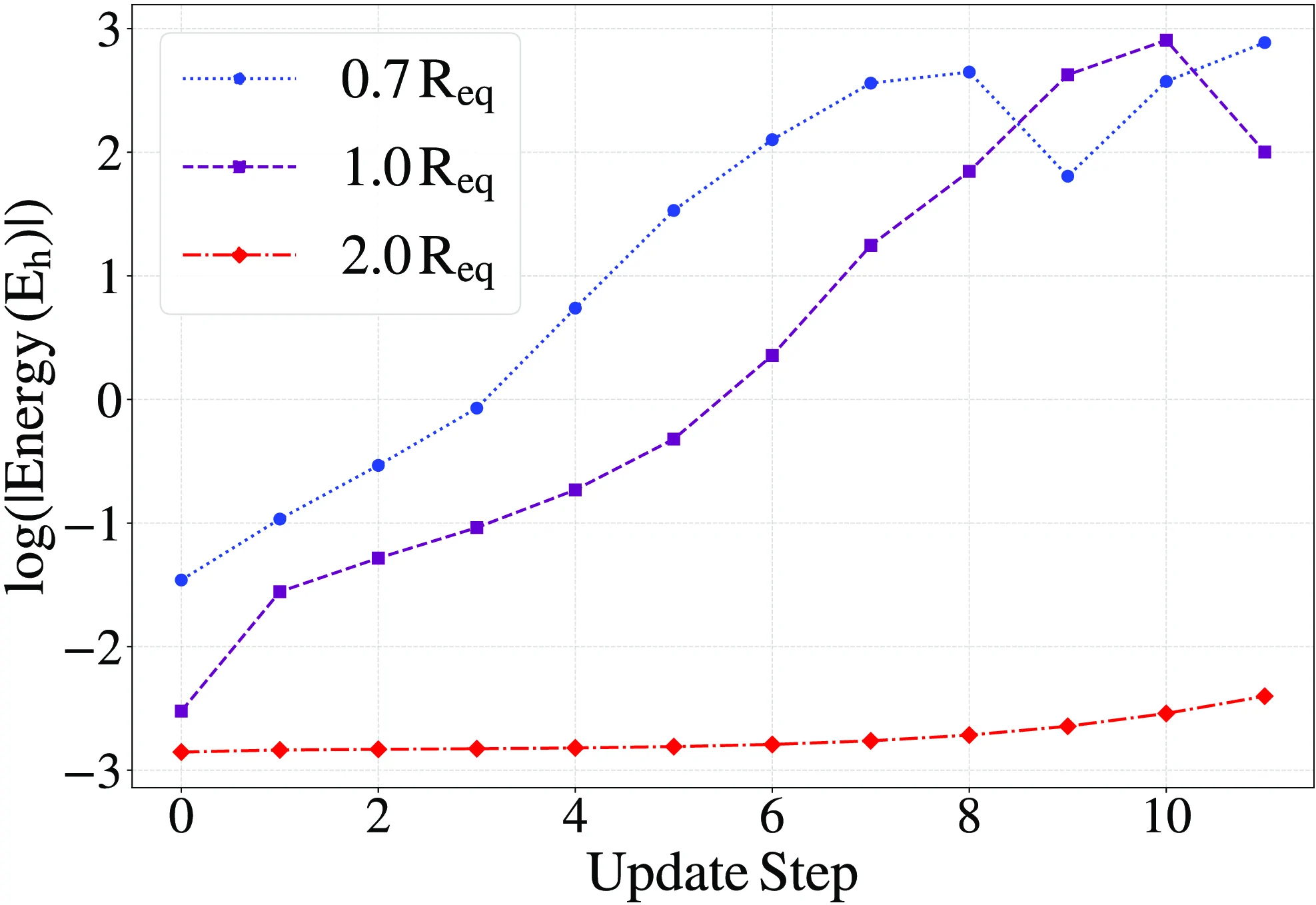
Convergence pattern (without including Ω^LM^ and *E*_LM_) of the *E*_int_^SART[HF]^ for H_2_O···H_2_O at *R*/*R*_eq_ = 0.7, 1.0, and 2.0, respectively. The logarithm of absolute value of *E*_int_^SART[HF]^, as a function of update step, is plotted. The *E*_int_^SART[HF]^ values are evaluated without including Ω^LM^ in the SART embedding potentials (Ω_B_^elst^ + Ω_B_^exch^), and hence also not adding *E*_LM_ to *E*_int_^SART[HF]^. The divergence of *E*_int_^SART[HF]^ over the range of intermonomer separations indicates the importance of the LM potentials and energy for the SART convergence.

## 5 Conclusions and Outlook

In this paper, we have introduced a new variational formalism for a self-consistent embedding while correctly taking into account exchange effects at all orders of the overlap matrix. This method, called symmetry-adapted relaxation theory, or SART, uses a nonorthogonal formalism to describe the self-consistent relaxation of each monomer in the field of its partner. Using a version of SART based on the Hartree–Fock formalism, we have shown that, at convergence, SART­[HF] reproduces the supermolecular Hartree–Fock interaction energy to a few thousands of a percent without explicit calculation of the supermolecular wave function. These tests were done using a dimer-centered basis set for monomers at close separation, which represents the worst-case scenario for conventional (point-charge) embedding techniques due to the polarization catastrophe. Additionally, the formalism of SART, particularly the use of nonorthogonal orbitals which retain monomer identity, is linked with that of symmetry-adapated perturbation theory, SAPT, and provides a means to extend the SAPT formalism to include infinite-order induction and the induction-dispersion coupling, something we are currently exploring in our groups.

We have tested the proof-of-principle implementation of SART based on Hartree–Fock, or SART­[HF], on strongly bound systems: H_2_O···H_2_O, Li^+^···H_2_O, and F^–^···H_2_O. Two of these show strong induction effects beyond second-order in the interaction operator, and with the SART algorithm we have been able to recover the infinite-order induction in these systems. On the other hand, in conventional SAPT theories we can only compute the induction to second or third order and have to recourse to supermolecular Hartree–Fock to estimate the remainder. We have shown that the construction of the induction energy in the SART formalism mirrors that of the polarization energy in classical polarization models, in that the induction energy contains a deformation term representing the energy penalty incurred in monomer relaxation, which is more than compensated by the energy of relaxation, leading to a net stabilization that gives the induction energy. However, SART differs from standard polarization models through the inclusion of all orders of response—linear and nonlinear—as well as charge-delocalization effects^90^ that are completely missing in classical polarization models as well as existing nonorthogonal theories like SCF-MI and BLW.^48–51^

One of the unexpected aspects of SART is the Landshoff and Murrell, or LM, contribution to the SART interaction energy and potential. The LM terms have previously been known to contribute to SAPT, but as they are zeroth-order in the interaction operator (at leading order), there has been a debate in the community whether they are part of the interaction energy. Here, we have shown that the LM terms have a crucial role as they help enforce the antisymmetry of the wave function in the energy expressions and the potential: without these terms we see a variational collapse of the SART monomer wave functions. Additionally, these terms contribute to the interaction energy through the monomer deformation energies. All of this highlights the importance of the LM terms in formulating a theory of embedding.

The present implementation of SART is necessarily limited computationally and theoretically as this paper was meant as a proof of the principle of the formalism. Now we lay out some of the outlook we have for SART that we are working on in our groups. Some of the numerical limitations are technical and have well-tested solutions which will be needed to extend SART to larger systems. These include convergence acceleration schemes for the SART updates, the use of parallelism, and schemes like density-fitting and formalisms based on atomic orbitals to avoid the 4-index integral transformation. Perhaps more importantly, there are theoretical improvements that can significantly extend the applicability of SART. First is the move to a density-matrix formulation of the theory that will be needed for SART based on a density-functional description of monomers, in analogy with SAPT­(DFT). Next, because SART yields symmetry-adapted, nonorthogonal monomer orbitals, it provides a natural entry point for the SAPT energy analysis: dispersion can be coupled consistently to induction and exchange–induction using the same orbital set, and frequency-dependent response (using linear-response time-dependent HF and DFT) can be integrated to treat polarization–dispersion coupling without double counting. The SART framework is inherently many-body: the Ω potentials generalize to trimers and higher clusters via incremental (*n*-body) expansions with a clear bookkeeping of Murrell–Landshoff and exchange terms, enabling scalable treatments of molecular clusters. Additional directions include analytic energy gradients for geometry optimizations and dynamics as well as open-shell variants. These extensions are also feasible as they mostly rely on the SCF know-how that is already established and widely implemented. With these developments, we aim to deliver through SART a variational and computationally efficient energy decomposition formalism for accurate interaction energies and mechanistic analysis across large molecular assemblies.

## Supplementary Material



## Appendix A. SART Potential Expressions

The form of the electrostatic potential is well-known in the literature^29^

30
$${\left(\Omega_{A}^{\mathrm{elst}}\right)}_{\mu }^{\nu }={\left(v_{A}\right)}_{\mu }^{\nu }+v_{\lambda \mu }^{\lambda \nu }$$

31
$${\left(\Omega_{B}^{\mathrm{elst}}\right)}_{\lambda }^{\kappa }={\left(v_{B}\right)}_{\lambda }^{\kappa }+v_{\lambda \mu }^{\kappa \mu }$$

We now consider the terms beyond electrostatics. In all expressions given below, we use the intermediates defined in our previous papers:^60,62^

32
$$A_{\beta_{1}}^{\beta }=\delta_{\beta_{1}}^{\beta }+S_{\beta_{1}}^{\alpha }S_{\alpha }^{\beta }+S_{\beta_{1}}^{\alpha }S_{\alpha }^{\beta_{2}}S_{\beta_{2}}^{\alpha_{1}}S_{\alpha_{1}}^{\beta }+...$$

33
$$B_{\alpha_{1}}^{\alpha }=\delta_{\alpha_{1}}^{\alpha }+S_{\beta }^{\alpha }S_{\alpha_{1}}^{\beta }+S_{\beta }^{\alpha }S_{\alpha_{2}}^{\beta }S_{\beta_{1}}^{\alpha_{2}}S_{\alpha_{1}}^{\beta_{1}}+...$$

$$C_{\rho_{1}}^{\rho }=\delta_{\rho_{1}}^{\rho }-S_{\beta }^{\rho }S_{\rho_{1}}^{\beta }-S_{\beta }^{\rho }S_{\alpha }^{\beta }S_{\beta_{1}}^{\alpha }S_{\rho_{1}}^{\beta_{1}}-...=\delta_{\rho_{1}}^{\rho }-S_{\beta }^{\rho }A_{\beta_{1}}^{\beta }S_{\rho_{1}}^{\beta_{1}}$$
34

35
$$D_{\sigma_{1}}^{\sigma }=\delta_{\sigma_{1}}^{\sigma }-S_{\alpha }^{\sigma }S_{\sigma_{1}}^{\alpha }-S_{\alpha }^{\sigma }S_{\beta }^{\alpha }S_{\alpha_{1}}^{\beta }S_{\sigma_{1}}^{\alpha_{1}}-...=\delta_{\sigma_{1}}^{\sigma }-S_{\alpha }^{\sigma }B_{\alpha_{1}}^{\alpha }S_{\sigma_{1}}^{\alpha_{1}}$$

36
$$E_{\alpha }^{\rho }=S_{\beta }^{\rho }S_{\alpha }^{\beta }+S_{\beta }^{\rho }S_{\alpha_{1}}^{\beta }S_{\beta_{1}}^{\alpha_{1}}S_{\alpha }^{\beta_{1}}+S_{\beta }^{\rho }S_{\alpha_{1}}^{\beta }S_{\beta_{1}}^{\alpha_{1}}S_{\alpha_{2}}^{\beta_{1}}S_{\beta_{2}}^{\alpha_{2}}S_{\alpha }^{\beta_{2}}+...=S_{\beta }^{\rho }A_{\beta_{1}}^{\beta }S_{\alpha }^{\beta_{1}}$$

37
$$F_{\beta }^{\sigma }=S_{\alpha }^{\sigma }S_{\beta }^{\alpha }+S_{\alpha }^{\sigma }S_{\beta_{1}}^{\alpha }S_{\alpha_{1}}^{\beta_{1}}S_{\beta }^{\alpha_{1}}+S_{\alpha }^{\sigma }S_{\beta_{1}}^{\alpha }S_{\alpha_{1}}^{\beta_{1}}S_{\beta_{2}}^{\alpha_{1}}S_{\alpha_{2}}^{\beta_{2}}S_{\beta }^{\alpha_{2}}+...=S_{\alpha }^{\sigma }B_{\alpha_{1}}^{\alpha }S_{\beta }^{\alpha_{1}}$$

38
$$G_{\rho }^{\sigma }=S_{\rho }^{\sigma }+S_{\alpha }^{\sigma }S_{\beta }^{\alpha }S_{\rho }^{\beta }+S_{\alpha }^{\sigma }S_{\beta }^{\alpha }S_{\alpha_{1}}^{\beta }S_{\beta_{1}}^{\alpha_{1}}S_{\rho }^{\beta_{1}}+...=S_{\sigma }^{\rho }+S_{\beta }^{\rho }A_{\beta_{1}}^{\beta }S_{\alpha }^{\beta_{1}}S_{\sigma }^{\alpha }$$

39
$$H_{\alpha }^{\beta }=S_{\alpha }^{\beta }+S_{\alpha_{1}}^{\beta }S_{\beta_{1}}^{\alpha_{1}}S_{\alpha }^{\beta_{1}}+S_{\alpha_{1}}^{\beta }S_{\beta_{1}}^{\alpha_{1}}S_{\alpha_{2}}^{\beta_{1}}S_{\beta_{2}}^{\alpha_{2}}S_{\alpha }^{\beta_{2}}+...=A_{\beta_{1}}^{\beta }S_{\alpha }^{\beta_{1}}=S_{\alpha_{1}}^{\beta }B_{\alpha }^{\alpha_{1}}$$

40
$$I_{\beta }^{\rho }=S_{\beta }^{\rho }+S_{\beta_{1}}^{\rho }S_{\alpha }^{\beta_{1}}S_{\beta }^{\alpha }+S_{\beta_{1}}^{\rho }S_{\alpha }^{\beta_{1}}S_{\beta_{2}}^{\alpha }S_{\alpha_{1}}^{\beta_{2}}S_{\beta }^{\alpha_{1}}+...=S_{\beta_{1}}^{\rho }A_{\beta }^{\beta_{1}}$$

41
$$J_{\alpha }^{\sigma }=S_{\alpha }^{\sigma }+S_{\alpha_{1}}^{\sigma }S_{\beta }^{\alpha_{1}}S_{\alpha }^{\beta }+S_{\alpha_{1}}^{\sigma }S_{\beta }^{\alpha_{1}}S_{\alpha_{2}}^{\beta }S_{\beta_{1}}^{\alpha_{2}}S_{\alpha }^{\beta_{1}}+...=S_{\alpha_{1}}^{\sigma }B_{\alpha }^{\alpha_{1}}$$

These intermediates follow from the analytic summation of a geometric series in overlap–matrix products. We also tested the *S* ^2^ and *S* ^4^ truncations for Ω_A_^exch^, Ω_B_^exch^, Ω_A_^LM^, and Ω_B_^LM^, which are obtained by truncating the corresponding series accordingly.

The general definition of the Omega-exchange matrix elements is

42
$$\begin{array}{c} (\Omega_{A}^{\mathrm{exch}}{)}_{\mu }^{\nu }=\frac{1}{2}\left\langle \Phi_{A}\Phi_{B}\right|\left\{b_{\mu },\left[\left\{V,P\right\},b_{\nu }^{†}\right]\right\}|\Phi_{A}\Phi_{B}\rangle_{L}\\ {\left(\Omega_{B}^{\mathrm{exch}}\right)}_{\lambda }^{\kappa }=\frac{1}{2}\left\langle \Phi_{A}\Phi_{B}\right|\left\{a_{\lambda },\left[\left\{V,P\right\},a_{\kappa }^{†}\right]\right\}|\Phi_{A}\Phi_{B}\rangle_{L} \end{array}$$

Below, we give the specific expressions only for the monomer B case. For the OO block of Ω_B_^exch^:

43
(ΩBexch)αα1=12⟨ΦAΦB|{aα,[{V,P},aα1†]}|ΦAΦB⟩L=12[(ΩBexch)α,VPα1+(ΩBexch)α,PVα1]

where the first term reads

44
$${\left(\Omega_{B}^{\mathrm{exch}}\right)}_{\alpha ,\mathrm{VP}}^{\alpha_{1}}={\left(\Omega_{B}^{\mathrm{elst}}\right)}_{\alpha_{2}}^{\alpha_{1}}B_{\alpha }^{\alpha_{2}}-{\left(\Omega_{B}^{\mathrm{elst}}\right)}_{\alpha_{2}}^{\rho }B_{\alpha }^{\alpha_{2}}E_{\rho }^{\alpha_{1}}-{\left(\Omega_{A}^{\mathrm{elst}}\right)}_{\beta }^{\sigma }H_{\alpha }^{\beta }J_{\sigma }^{\alpha_{1}}-v_{\alpha_{2}\beta }^{\rho \sigma }B_{\alpha }^{\alpha_{2}}I_{\rho }^{\beta }J_{\sigma }^{\alpha_{1}}-v_{\alpha_{2}\beta }^{\rho \sigma }E_{\rho }^{\alpha_{1}}H_{\alpha }^{\beta }J_{\sigma }^{\alpha_{2}}+v_{\alpha_{2}\beta }^{\rho \sigma }E_{\rho }^{\alpha_{2}}H_{\alpha }^{\beta }J_{\sigma }^{\alpha_{1}}+v_{\alpha_{2}\beta }^{\rho \sigma }B_{\alpha }^{\alpha_{2}}E_{\rho }^{\alpha_{1}}F_{\sigma }^{\beta }+v_{\alpha_{2}\beta }^{\alpha_{1}\sigma }H_{\alpha }^{\beta }J_{\sigma }^{\alpha_{2}}-v_{\alpha_{2}\beta }^{\alpha_{1}\sigma }B_{\alpha }^{\alpha_{2}}F_{\sigma }^{\beta }-{\left(\Omega_{B}^{\mathrm{elst}}\right)}_{\alpha }^{\alpha_{1}}$$

The second term in eq 43 is the respective matrix element of the Hermitian conjugate of the matrix defined by the first term spanned in the OO space. For the case of real orbitals, (Ω_B_^exch^)_α,PV_^α_1_^ = (Ω_B_^exch^)_α_1_,VP_^α^. As we will be dealing with only real orbitals, this symmetry also holds in case of the OO and VV blocks of Ω^LM^.

Similarly, for the VV block of Ω_B_^exch^:

45
(ΩBexch)ρρ1=12⟨ΦAΦB|{aρ,[{V,P},aρ1†]}|ΦAΦB⟩L=12[(ΩBexch)ρ,VPρ1+(ΩBexch)ρ,PVρ1]

where the first term is

46
$${\left(\Omega_{B}^{\mathrm{exch}}\right)}_{\rho ,\mathrm{VP}}^{\rho_{1}}={\left(\Omega_{B}^{\mathrm{elst}}\right)}_{\rho }^{\rho_{2}}C_{\rho_{2}}^{\rho_{1}}+{\left(\Omega_{B}^{\mathrm{elst}}\right)}_{\rho_{2}}^{\alpha }C_{\rho }^{\rho_{2}}E_{\alpha }^{\rho_{1}}-{\left(\Omega_{A}^{\mathrm{elst}}\right)}_{\beta }^{\sigma }G_{\sigma }^{\rho_{1}}I_{\rho }^{\beta }-v_{\alpha \beta }^{\rho_{2}\sigma }E_{\rho }^{\alpha }G_{\sigma }^{\rho_{1}}I_{\rho_{2}}^{\beta }+v_{\alpha \beta }^{\rho_{2}\sigma }C_{\rho_{2}}^{\rho_{1}}I_{\rho }^{\beta }J_{\sigma }^{\alpha }-v_{\alpha \beta }^{\rho_{2}\sigma }C_{\rho_{2}}^{\rho_{1}}E_{\rho }^{\alpha }F_{\sigma }^{\beta }+v_{\alpha \beta }^{\rho_{2}\sigma }E_{\rho_{2}}^{\alpha }G_{\sigma }^{\rho_{1}}I_{\rho }^{\beta }-v_{\rho \beta }^{\rho_{2}\sigma }C_{\rho_{2}}^{\rho_{1}}F_{\sigma }^{\beta }-v_{\rho \beta }^{\rho_{2}\sigma }G_{\sigma }^{\rho_{1}}I_{\rho_{2}}^{\beta }-{\left(\Omega_{B}^{\mathrm{elst}}\right)}_{\rho }^{\rho_{1}}$$

For the OV and VO blocks of Ω_B_^exch^, we have

$${\left(\Omega_{B}^{\mathrm{exch}}\right)}_{\alpha }^{\rho }={\left(\Omega_{B}^{\mathrm{exch}}\right)}_{\rho }^{\alpha }=B_{\alpha }^{\alpha_{1}}C_{\rho_{1}}^{\rho }{\left(\Omega_{B}^{\mathrm{elst}}\right)}_{\alpha_{1}}^{\rho_{1}}+G_{\sigma }^{\rho }H_{\alpha }^{\beta }{\left(\Omega_{A}^{\mathrm{elst}}\right)}_{\beta }^{\sigma }+C_{\rho_{1}}^{\rho }H_{\alpha }^{\beta }J_{\sigma }^{\alpha_{1}}v_{\alpha_{1}\beta }^{\rho_{1}\sigma }-I_{\rho_{1}}^{\beta }B_{\alpha }^{\alpha_{1}}G_{\sigma }^{\rho }v_{\alpha_{1}\beta }^{\rho_{1}\sigma }+E_{\rho_{1}}^{\alpha_{1}}G_{\sigma }^{\rho }H_{\alpha }^{\beta }v_{\alpha_{1}\beta }^{\rho_{1}\sigma }-B_{\alpha }^{\alpha_{1}}C_{\rho_{1}}^{\rho }F_{\sigma }^{\beta }v_{\alpha_{1}\beta }^{\rho_{1}\sigma }+E_{\alpha_{1}}^{\rho }{\left(\Omega_{B}^{\mathrm{elst}}\right)}_{\alpha }^{\alpha_{1}}-E_{\alpha }^{\rho_{1}}{\left(\Omega_{B}^{\mathrm{elst}}\right)}_{\rho_{1}}^{\rho }-E_{\alpha }^{\rho_{1}}E_{\alpha_{1}}^{\rho }{\left(\Omega_{B}^{\mathrm{elst}}\right)}_{\rho_{1}}^{\alpha_{1}}-I_{\beta }^{\rho }J_{\alpha }^{\sigma }{\left(\Omega_{A}^{\mathrm{elst}}\right)}_{\sigma }^{\beta }+I_{\beta }^{\rho }J_{\alpha_{1}}^{\sigma }v_{\alpha \sigma }^{\alpha_{1}\beta }-I_{\beta }^{\rho_{1}}J_{\alpha }^{\sigma }v_{\rho_{1}\sigma }^{\rho \beta }+E_{\alpha }^{\rho_{1}}F_{\beta }^{\sigma }v_{\rho_{1}\sigma }^{\rho \beta }-E_{\alpha_{1}}^{\rho }F_{\beta }^{\sigma }v_{\alpha \sigma }^{\alpha_{1}\beta }+E_{\alpha }^{\rho_{1}}E_{\alpha_{1}}^{\rho }F_{\beta }^{\sigma }v_{\rho_{1}\sigma }^{\alpha_{1}\beta }+E_{\alpha_{1}}^{\rho_{1}}I_{\beta }^{\rho }J_{\alpha }^{\sigma }v_{\rho_{1}\sigma }^{\alpha_{1}\beta }-E_{\alpha_{1}}^{\rho }I_{\beta }^{\rho_{1}}J_{\alpha }^{\sigma }v_{\rho_{1}\sigma }^{\alpha_{1}\beta }-E_{\alpha }^{\rho_{1}}I_{\beta }^{\rho }J_{\alpha_{1}}^{\sigma }v_{\rho_{1}\sigma }^{\alpha_{1}\beta }-F_{\beta }^{\sigma }v_{\alpha \sigma }^{\rho \beta }-{\left(\Omega_{B}^{\mathrm{elst}}\right)}_{\alpha }^{\rho }$$
47

The expressions for the Landshoff–Murrell potential will be given below:

48
$$\begin{array}{c} {\left(\Omega_{A}^{\mathrm{LM}}\right)}_{\mu }^{\nu }=\frac{1}{2}\left\langle \Phi_{A}\Phi_{B}\right|\left\{b_{\mu },\left[\left\{H_{A}+H_{B},P\right\},b_{\nu }^{†}\right]\right\}|\Phi_{A}\Phi_{B}\rangle_{L}\\ {\left(\Omega_{B}^{\mathrm{LM}}\right)}_{\lambda }^{\kappa }=\frac{1}{2}\left\langle \Phi_{A}\Phi_{B}\right|\left\{a_{\lambda },\left[\left\{H_{A}+H_{B},P\right\},a_{\kappa }^{†}\right]\right\}|\Phi_{A}\Phi_{B}\rangle_{L} \end{array}$$

The Ω_A_^LM^ potential can be split as Ω_A_^L^ + Ω_A_^M^ as follows.

49
$$\begin{array}{c} {\left(\Omega_{A}^{L}\right)}_{\mu }^{\nu }=\frac{1}{2}\left\langle \Phi_{A}\Phi_{B}\right|\left\{b_{\mu },\left[\left\{F_{A}+F_{B},P\right\},b_{\nu }^{†}\right]\right\}|\Phi_{A}\Phi_{B}\rangle_{L}\\ {\left(\Omega_{A}^{M}\right)}_{\mu }^{\nu }=\frac{1}{2}\left\langle \Phi_{A}\Phi_{B}\right|\left\{b_{\mu },\left[\left\{W_{A}+W_{B},P\right\},b_{\nu }^{†}\right]\right\}|\Phi_{A}\Phi_{B}\rangle_{L} \end{array}$$

Similarly, for Ω_B_^LM^,

50
$$\begin{array}{c} {\left(\Omega_{B}^{L}\right)}_{\lambda }^{\kappa }=\frac{1}{2}\left\langle \Phi_{A}\Phi_{B}\right|\left\{a_{\lambda },\left[\left\{F_{A}+F_{B},P\right\},a_{\kappa }^{†}\right]\right\}|\Phi_{A}\Phi_{B}\rangle_{L}\\ {\left(\Omega_{B}^{M}\right)}_{\lambda }^{\kappa }=\frac{1}{2}\left\langle \Phi_{A}\Phi_{B}\right|\left\{a_{\lambda },\left[\left\{W_{A}+W_{B},P\right\},a_{\kappa }^{†}\right]\right\}|\Phi_{A}\Phi_{B}\rangle_{L} \end{array}$$

For the OO block of Ω_B_^L^, we have

51
(ΩBL)αα1=12⟨ΦAΦB|{aα,[{FA+FB,P},aα1†]}|ΦAΦB⟩L=12[(ΩBL)α,FPα1+(ΩBL)α,PFα1]

52
$${\left(\Omega_{B}^{L}\right)}_{\alpha ,\mathrm{FP}}^{\alpha_{1}}={\left(F_{A}\right)}_{\alpha_{2}}^{\alpha_{1}}B_{\alpha }^{\alpha_{2}}-{\left(F_{A}\right)}_{\alpha_{2}}^{\rho }B_{\alpha }^{\alpha_{2}}E_{\rho }^{\alpha_{1}}-{\left(F_{A}\right)}_{\alpha }^{\alpha_{1}}-{\left(F_{B}\right)}_{\beta }^{\sigma }H_{\alpha }^{\beta }J_{\sigma }^{\alpha_{1}}$$

Here, we note that (Ω_B_^L^)_α,*FP*_^α_1_^ = (Ω_B_^L^)_α_1_,*PF*_^α^.

Summing up, we can write

53
$${\left(\Omega_{B}^{L}\right)}_{\alpha }^{\alpha_{1}}=\frac{1}{2}\left[{\left(F_{A}\right)}_{\alpha_{2}}^{\alpha_{1}}B_{\alpha }^{\alpha_{2}}+{\left(F_{A}\right)}_{\alpha }^{\alpha_{2}}B_{\alpha_{2}}^{\alpha_{1}}-{\left(F_{A}\right)}_{\alpha_{2}}^{\rho }B_{\alpha }^{\alpha_{2}}E_{\rho }^{\alpha_{1}}-{\left(F_{A}\right)}_{\rho }^{\alpha_{2}}B_{\alpha_{2}}^{\alpha_{1}}E_{\alpha }^{\rho }-{\left(F_{B}\right)}_{\beta }^{\sigma }H_{\alpha }^{\beta }J_{\sigma }^{\alpha_{1}}-{\left(F_{B}\right)}_{\sigma }^{\beta }H_{\beta }^{\alpha_{1}}J_{\alpha }^{\sigma }\right]-{\left(F_{A}\right)}_{\alpha }^{\alpha_{1}}$$

Similarly, for the VV block of Ω_B_^L^:

54
$${\left(\Omega_{B}^{L}\right)}_{\rho }^{\rho_{1}}=\frac{1}{2}\left[{\left(F_{A}\right)}_{\rho }^{\rho_{2}}C_{\rho_{2}}^{\rho_{1}}+{\left(F_{A}\right)}_{\rho_{2}}^{\rho_{1}}C_{\rho }^{\rho_{2}}+{\left(F_{A}\right)}_{\rho_{2}}^{\alpha }C_{\rho }^{\rho_{2}}E_{\alpha }^{\rho_{1}}+{\left(F_{A}\right)}_{\alpha }^{\rho_{2}}C_{\rho_{2}}^{\rho_{1}}E_{\rho }^{\alpha }-{\left(F_{B}\right)}_{\beta }^{\sigma }G_{\sigma }^{\rho_{1}}I_{\rho }^{\beta }-{\left(F_{B}\right)}_{\sigma }^{\beta }G_{\rho }^{\sigma }I_{\beta }^{\rho_{1}}\right]-{\left(F_{A}\right)}_{\rho }^{\rho_{1}}$$

For the OV and VO blocks of Ω_B_^L^, we obtain

55
(ΩBL)αρ=(ΩBL)ρα=12[(FA)α1ρ1Bαα1Cρ1ρ−(FA)ρ1α1Eαρ1Eα1ρ+(FA)αα1Eα1ρ−(FA)ρ1ρEαρ1−(FB)βσGσρHαβ−(FB)σβIβρJασ−(FA)αρ]

Next, for the Murrell potential terms, let us first define the *W*_A_, *W*_B_ operators.

56
WA=HA−FA=14(vκκ1λλ1−vκκ1λ1λ)aλλ1κκ1−(vκαλα−vκααλ)aλκ=14Wκκ1λλ1aλλ1κκ1−Wκαλαaλκ

where *W*_κκ_1__^*λλ*_1_^ = (*v*_κκ_1__^λλ_1_^ – *v*_κκ_1__^λ_1_λ^).

For the OO block of Ω_B_^M^, eq 50, we have

57
$${\left(\Omega_{B}^{M}\right)}_{\alpha }^{\alpha_{1}}=\frac{1}{2}\left[W_{\alpha_{2}\alpha_{3}}^{\alpha_{1}\rho }E_{\rho }^{\alpha_{2}}B_{\alpha }^{\alpha_{3}}-W_{\alpha_{2}\alpha_{3}}^{\rho \rho_{1}}E_{\rho }^{\alpha_{3}}E_{\rho_{1}}^{\alpha_{1}}B_{\alpha }^{\alpha_{2}}-W_{\beta \beta_{1}}^{\sigma \sigma_{1}}F_{\sigma_{1}}^{\beta }H_{\alpha }^{\beta_{1}}J_{\sigma }^{\alpha_{1}}+W_{\alpha \rho }^{\alpha_{2}\alpha_{3}}E_{\alpha_{2}}^{\rho }B_{\alpha_{3}}^{\alpha_{1}}-W_{\rho \rho_{1}}^{\alpha_{2}\alpha_{3}}E_{\alpha_{3}}^{\rho }E_{\alpha }^{\rho_{1}}B_{\alpha_{2}}^{\alpha_{1}}-W_{\sigma \sigma_{1}}^{\beta \beta_{1}}F_{\beta }^{\sigma_{1}}H_{\beta_{1}}^{\alpha_{1}}J_{\alpha }^{\sigma }\right]$$

The VV block of Ω_B_^M^ is given by

58
$${\left(\Omega_{B}^{M}\right)}_{\rho }^{\rho_{1}}=\frac{1}{2}\left[W_{\rho \alpha_{1}}^{\rho_{2}\rho_{3}}C_{\rho_{3}}^{\rho_{1}}E_{\rho_{2}}^{\alpha_{1}}+W_{\alpha \alpha_{1}}^{\rho_{2}\rho_{3}}C_{\rho_{2}}^{\rho_{1}}E_{\rho_{3}}^{\alpha }E_{\rho }^{\alpha_{1}}-W_{\beta \beta_{1}}^{\sigma \sigma_{1}}F_{\sigma_{1}}^{\beta }G_{\sigma }^{\rho_{1}}I_{\rho }^{\beta_{1}}+W_{\rho_{2}\rho_{3}}^{\rho_{1}\alpha }C_{\rho }^{\rho_{3}}E_{\alpha }^{\rho_{2}}+W_{\rho_{2}\rho_{3}}^{\alpha \alpha_{1}}C_{\rho }^{\rho_{2}}E_{\alpha }^{\rho_{3}}E_{\alpha_{1}}^{\rho_{1}}-W_{\sigma \sigma_{1}}^{\beta \beta_{1}}F_{\beta }^{\sigma_{1}}G_{\rho }^{\sigma }I_{\beta_{1}}^{\rho_{1}}\right]$$

Finally, the OV and VO blocks of Ω_B_^M^ are

59
(ΩBM)ρα=(ΩBM)αρ=12[Wα1α2ρ1ρ2Bαα1Cρ2ρEρ1α2+Wαρ1α1ρEα1ρ1+Wαρ1α1α2Eα1ρ1Eα2ρ−Wρ1ρ2α1ρEαρ1Eα1ρ2−Wρ1ρ2α1α2Eαρ2Eα1ρEα2ρ1−Wββ1σσ1Fσβ1Gσ1ρHαβ−Wσσ1ββ1Fβ1σIβρJασ1]

## Notes

Geometries used in the calculations, convergence patterns and components for long-range region of studied complexes (PDF)
