# Supplementary material for: Symmetry-Adapted Relaxation Theory (SART): Variational Embedding for Convergent Infinite-Order Induction without Overpolarization
Source: J Chem Theory Comput. 2026 Aug 13;22(16):8239–56. doi: 10.1021/acs.jctc.6c00770 (PMC13523646; doi:10.1021/acs.jctc.6c00770)
Supplement: Supplementary file 1 [file ct6c00770_si_001.pdf]

Supplementary Information for the article: Symmetry-Adapted Relaxation Theory (SART): variational embedding for convergent infinite-order induction without overpolarization

Humahuti Dihingia,† Bartosz Tyrcha,† Edoardo Vanich,‡ Konrad Patkowski,¶ Alston J. Misquitta,*,§ and Piotr S Żuchowski *,†

†*Institute of Physics, Faculty of Physics, Astronomy and Informatics, Nicolaus Copernicus University in Toruń, Grudziądzka 5/7, 87-100 Toruń, Poland*

‡*Sorbonne Université, Laboratoire de Chimie Théorique, UMR 7616 CNRS, 75005 Paris, France*

¶*Department of Chemistry and Biochemistry, Auburn University, Auburn, Alabama 36849, USA*

§*Department of Physics and Astronomy, Queen Mary, University of London, London E1 4NS, United Kingdom*

E-mail: a.j.misquitta@qmul.ac.uk; pzuch@fizyka.umk.pl

Contents

1 Convergence pattern of SART **S2**

2 Table of SART and SAPT components S4

3 Geometries at equilibrium S5

3.1 $H_2O \cdots H_2O$ S5

3.2 $Li^+ \cdots H_2O$ S5

3.3 $F^- \cdots H_2O$ S5

This document includes the additional figures, table to the main text, and equilibrium geometries of the studied systems.

1 Convergence pattern of SART

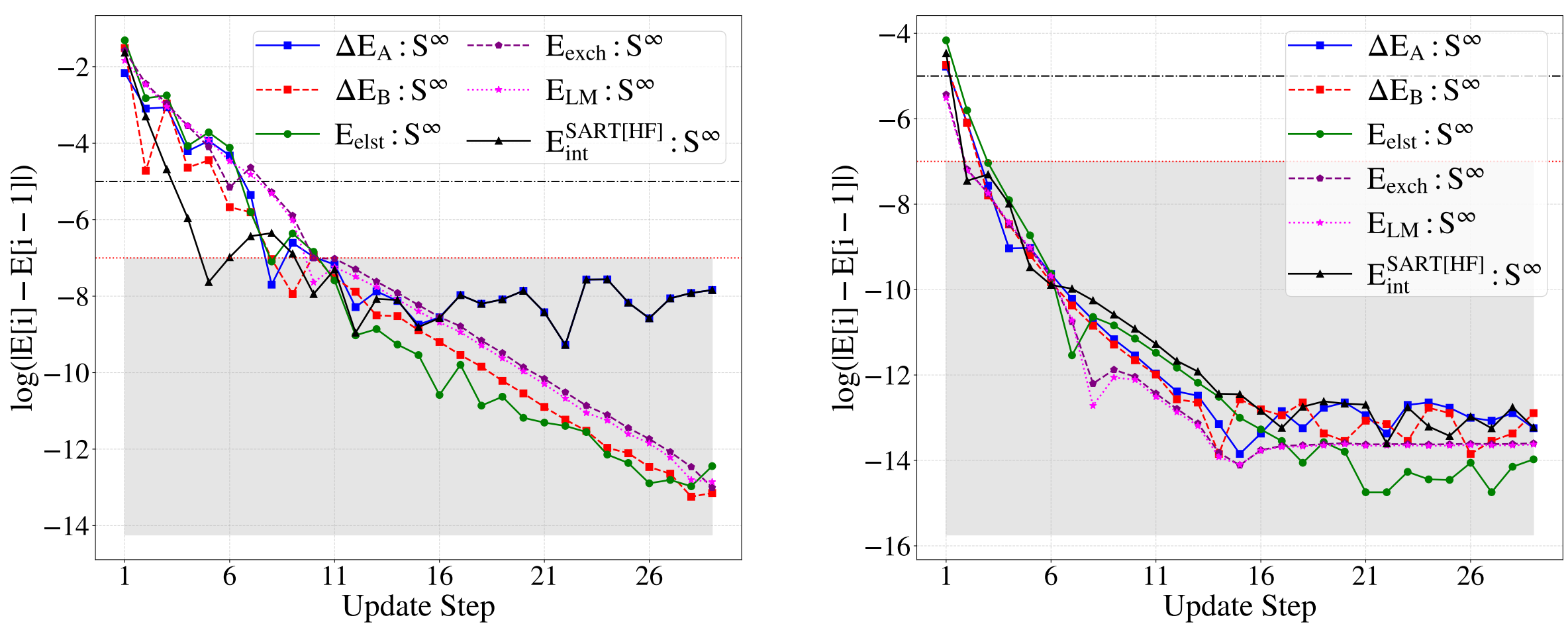

(a) 0.7 R_{eq} (b) 2.0 R_{eq}

Figure S1: Convergence pattern of $E_{int}^{SART[HF]}$ and its primary components for $H_2O \cdots H_2O$ with the full Ω^{exch} and Ω^{LM} potentials at $R/R_{eq} = 0.7$, 2.0, respectively. In each plot, the logarithms of the absolute differences between consecutive energy values are plotted. The black horizontal line represents the reference accuracy of interaction energy, 10^{-5} E_h and the red dotted line represents the convergence threshold, 10^{-7} E_h for the SART scheme. The convergence for $E_{int}^{SART[HF]}$ is achieved using self-consistent relaxation after update step 5, 2 in S1a, S1b, respectively.

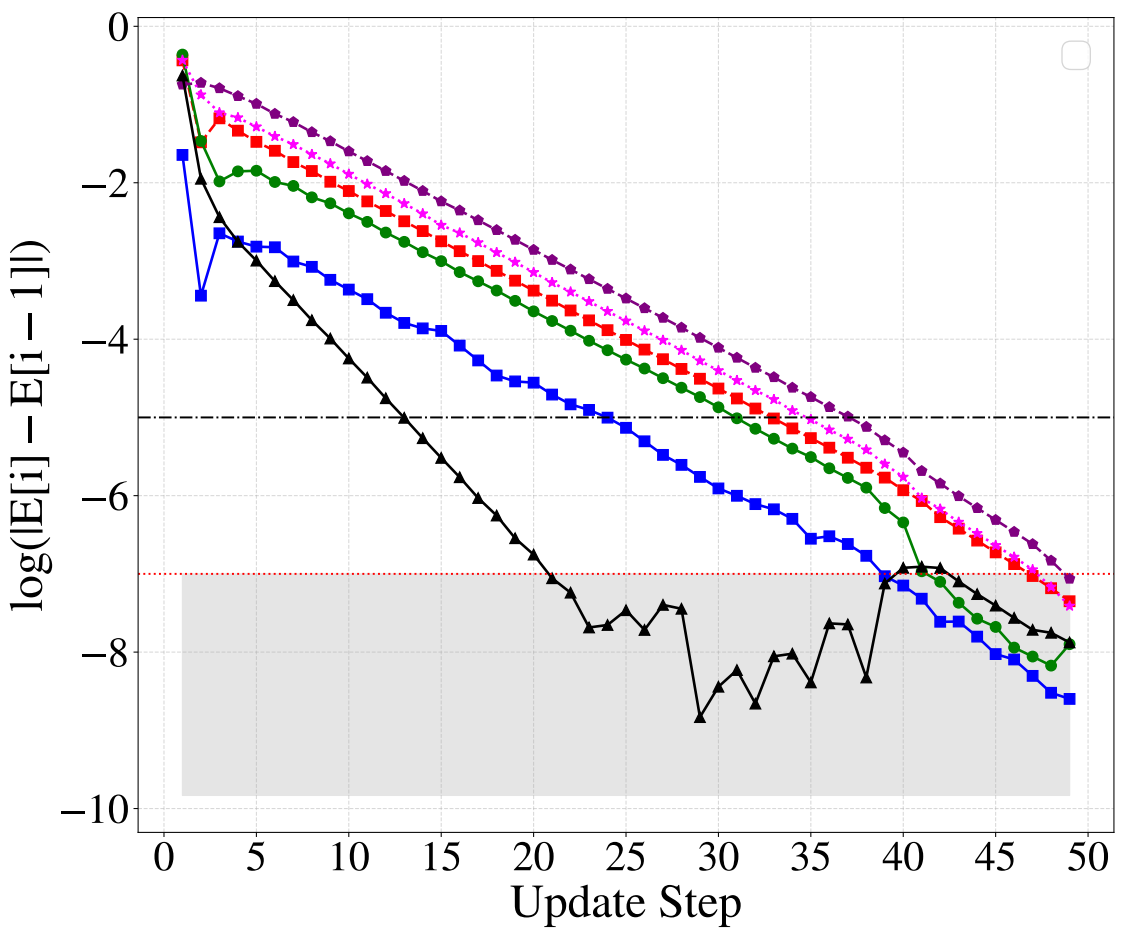

(a) 0.5 R_{eq}

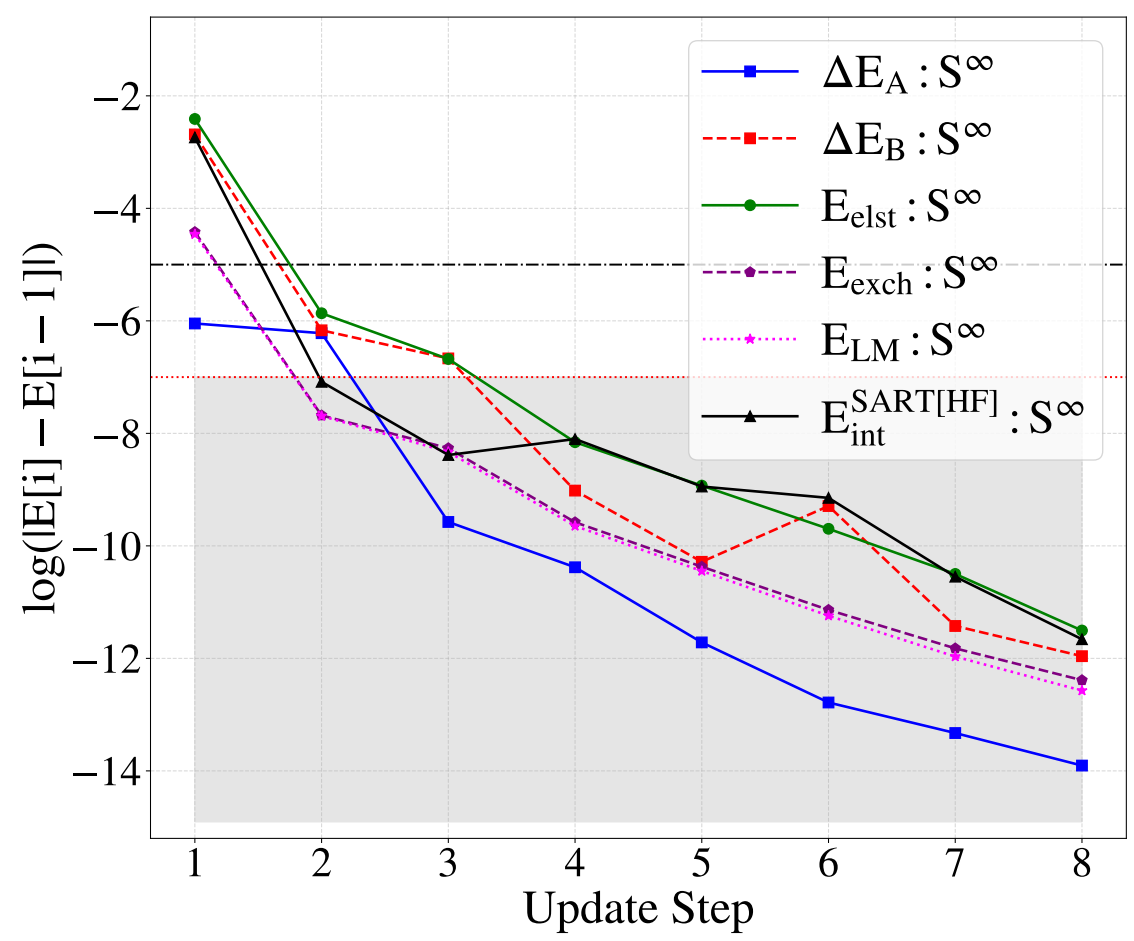

(b) 2.0 R_{eq}

Figure S2: Convergence pattern of $E_{int}^{SART[HF]}$ and its primary components for $Li^+ \cdots H_2O$ with the full Ω^{exch} and Ω^{LM} potentials at $R/R_{eq} = 0.5, 1.0, 2.0$, respectively. In each plot, the logarithms of the absolute differences between consecutive energy values are plotted. The black horizontal line represents the reference accuracy of interaction energy, 10^{-5} E_h and the red dotted line represents the convergence threshold, 10^{-7} E_h for the SART scheme. The convergence for $E_{int}^{SART[HF]}$ is achieved using SART self-consistent scheme after update step 21 and 2 in S2a, S2b, respectively.

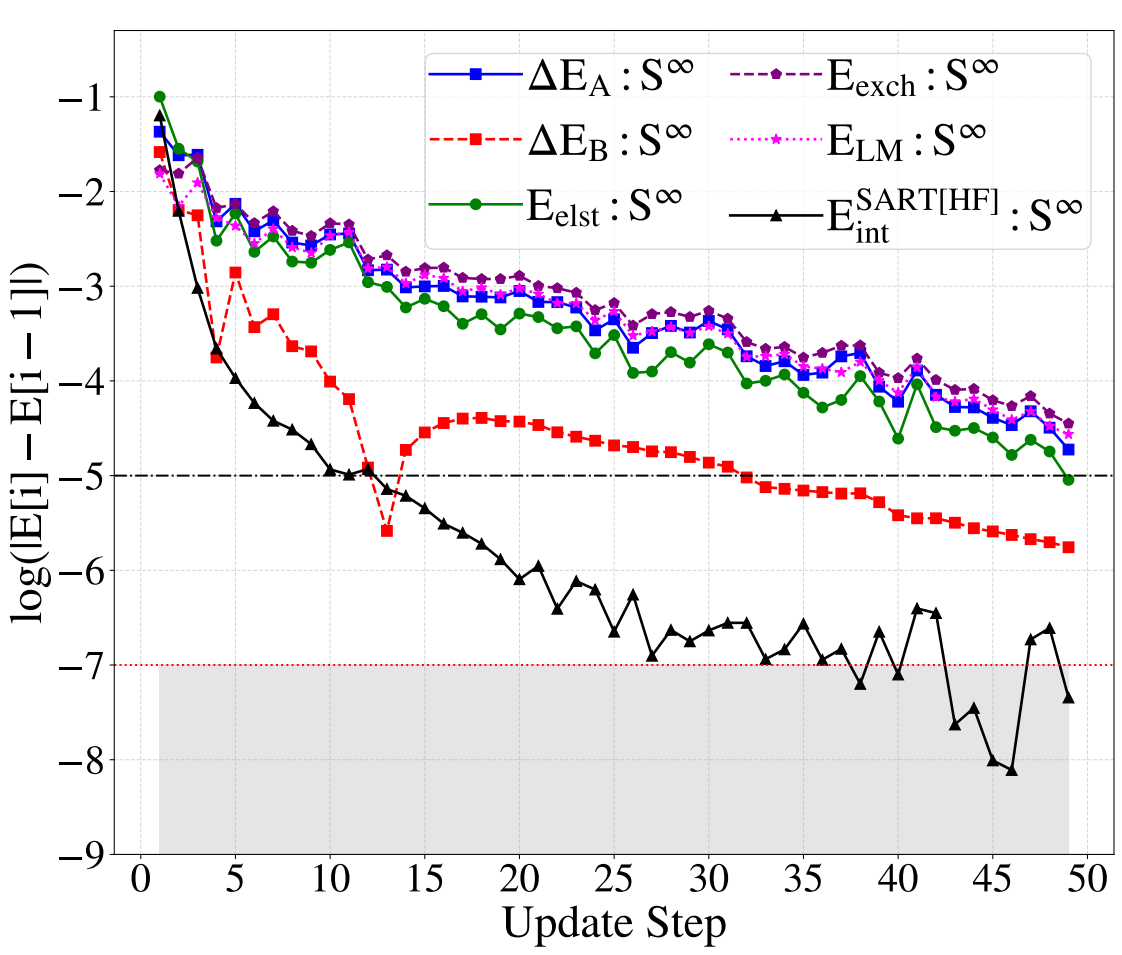

(a) 0.9 R_{eq}

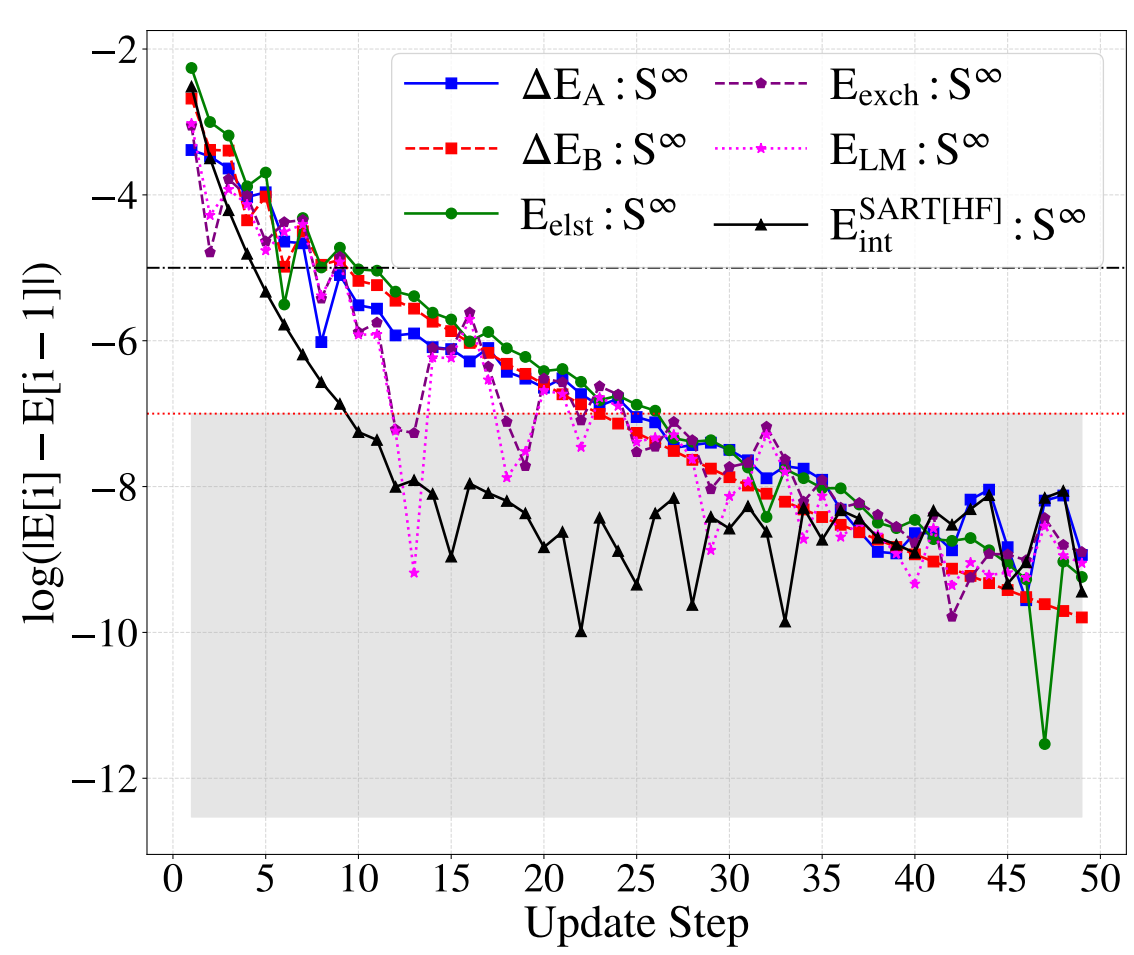

(b) 2.0 R_{eq}

Figure S3: Convergence pattern of $E_{int}^{SART[HF]}$ and its primary components for $F^- \cdots H_2O$ with the full Ω^{exch} and Ω^{LM} potentials at $R/R_{eq} = 0.9, 2.0$, respectively. In each plot, the logarithms of the absolute differences between consecutive energy values are plotted. The black horizontal line represents the reference accuracy of interaction energy, 10^{-5} E_h and the red dotted line represents the convergence threshold, 10^{-7} E_h for the SART scheme. The convergence for $E_{int}^{SART[HF]}$ is achieved using SART diagonal relaxation scheme after update step 38 and 10 in S3a, S3b, respectively.

2 Table of SART and SAPT components

Table S1: The SART[HF] energy contributions in comparison to the SAPT0 energies for $H_2O \cdots H_2O$, $Li^+ \cdots H_2O$, and $F^- \cdots H_2O$ at $R/R_{eq} = 2.0$. All energy values are in millihartree (mE_h). The distances are between $H \cdots O$, $Li^+ \cdots O$ and $F^- \cdots H$, respectively.

System	$H_2O \cdots H_2O$	$Li^+ \cdots H_2O$	$F^- \cdots H_2O$
Distance (Å)	4.023	3.740	3.811
SAPT0 interaction energy components:			
$E^{(10)}_{elst}$	−1.408	−11.630	−16.891
$E^{(10)}_{exch}$	4.874×10^{-3}	1.240×10^{-2}	0.821
$E^{(20)}_{IND}$	-3.307×10^{-2}	−1.695	−3.300
$E^{(20)}_{ind,resp}$	-3.371×10^{-2}	−1.712	−3.607
$E^{(20)}_{exch-ind,resp}$	6.454×10^{-4}	1.759×10^{-2}	0.307
SART primary components:			
ΔE_A	1.736×10^{-2}	1.502×10^{-3}	1.231
ΔE_B	1.900×10^{-2}	2.061	3.067
E_{LM}	3.000×10^{-3}	3.476×10^{-2}	−0.715
$E_{L,A}$	-1.025×10^{-3}	3.723×10^{-5}	0.646
$E_{M,A}$	7.365×10^{-8}	4.685×10^{-8}	1.419×10^{-3}
$E_{L,B}$	4.025×10^{-3}	3.472×10^{-2}	−1.364
$E_{M,B}$	8.232×10^{-8}	2.904×10^{-8}	1.275×10^{-3}
E_{elst}	−1.478	−15.514	−24.422
E_{exch}	1.258×10^{-3}	-2.553×10^{-2}	1.314
SART physical components:			
$E^{[0]}_{elst}$	−1.408	−11.630	−16.891
$E^{[0]}_{exch}$	4.874×10^{-3}	1.240×10^{-2}	0.821
E_{ind}	-3.422×10^{-2}	−1.824	−3.456
E_{def}	3.935×10^{-2}	2.097	3.583
$E_{def,A}$	1.633×10^{-2}	1.539×10^{-3}	1.878
$E_{def,B}$	2.302×10^{-2}	2.096	1.705
E_{rel}	-7.357×10^{-2}	−3.921	−7.038
ΔE_{elst}	-6.996×10^{-2}	−3.883	−7.531
ΔE_{exch}	-3.616×10^{-3}	-3.792×10^{-2}	0.493
E^{SAPT0}_{int} (no disp)	−1.436	−13.312	−19.370
E^{HF}_{int}	−1.437	−13.442	−19.526
E^{SART}_{int}	−1.437	−13.442	−19.526
Rel. error(%)	0.004 157	0.000 332	0.001 318

3 Geometries at equilibrium

The unit presented here is Å.

3.1 $H_2O \cdots H_2O$

O	-0.70219605	-0.05606026	0.00994226
H	-1.02219322	0.84677578	-0.01148871
H	0.25752106	0.04212150	0.00521900
O	2.26888078	0.02634010	0.00050803
H	2.64550240	-0.41203997	0.76663241
H	2.64114510	-0.44987287	-0.74489447

3.2 $Li^+ \cdots H_2O$

Li	0.00000000	-2.16118751	3.05235459
O	0.00000000	-1.08059376	1.52617729
H	0.75668992	-0.49470182	1.52617729
H	-0.75668992	-0.49470182	1.52617729

3.3 $F^- \cdots H_2O$

F	-1.23638900	0.01223900	0.00000000
O	1.19756600	-0.10808700	0.00000000
H	1.41539700	0.82701400	0.00000000
H	0.13483000	-0.08437800	0.00000000
